# Supplementary material for: Transcriptomic Signature and PROTAC Strategy Revealed Histone Lysine Demethylase as a Target of Anticancer Activity of Deferiprone
Source: ACS Omega. 2026 Apr 28;11(18):26421–37. doi: 10.1021/acsomega.5c11926 (PMC13177236; doi:10.1021/acsomega.5c11926)
Supplement: Supplementary file 1 [file ao5c11926_si_001.pdf]

Transcriptomic Signature and PROTAC Strategy Revealed Histone Lysine Demethylase as a Target of Anticancer Activity of Deferiprone.

Alexis Johnston[1¶], Jeremiah O. Olugbami[1¶], Dipak Walunj[1¶], Arvind Bangaru,[2¶] Bocheng Wu[1], Ryan Kern,[1] Ruiqiao Yang,[2] Travis J. Nelson,[3] Brandon J. Clarke,[3] Janani Murugan[1], Nathaniel A Hathaway,[3] Yuhong Fan,[2, 4] Adegboyega K. Oyelere[1, 4*]

[1]School of Chemistry and Biochemistry, Georgia Institute of Technology, Atlanta, GA 30332-0400, USA; [2]School of Biological Sciences, Georgia Institute of Technology, Atlanta, GA, 30332-0400, USA; [3]The University of North Carolina Eshelman School of Pharmacy, Chapel Hill, NC, 27599, USA; [4]Parker H. Petit Institute for Bioengineering and Bioscience, Georgia Institute of Technology, Atlanta, GA 30332-0400, USA.

[*]Address correspondence to: Adegboyega K. Oyelere, PhD at email: aoyelere@gatech.edu or phone: 404-894-4047.

[¶]These authors contributed equally to the manuscript.

Keywords: Histone lysine demethylase; Deferiprone; RNA seq; PROTAC.

Supporting information

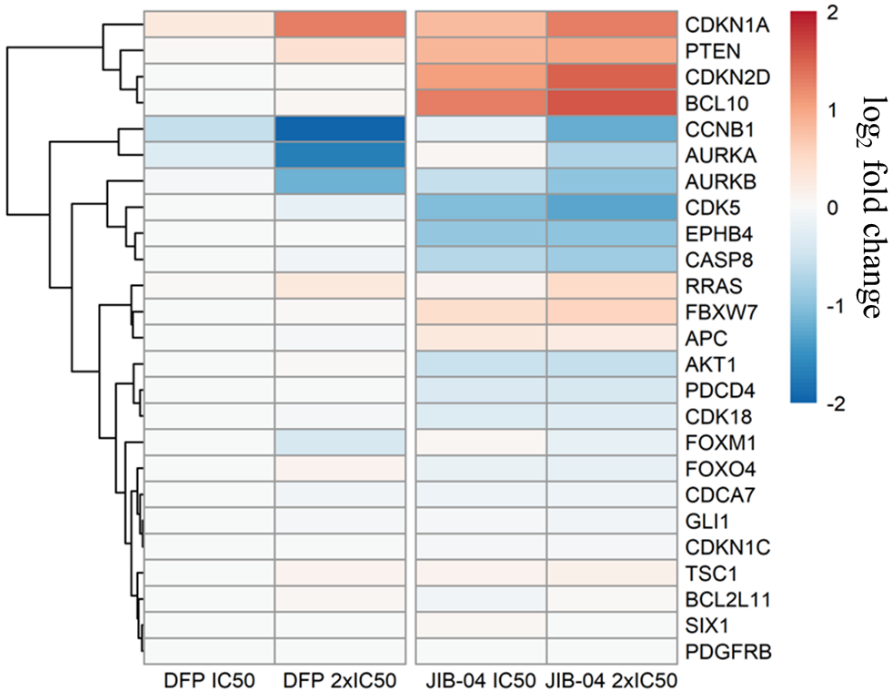

Figure S1. Effects of DFP and JIB-04 on genes involved in KDM inhibition in MCF-7 cells. Log2 fold change heatmap of genes significant to JIB-04 inhibition in Ewing Sarcoma displaying upregulation of CDKN1A (p21) by DFP 2x IC_{50} (log2 fold change = +1.3) and JIB-04 2x IC_{50} (log2 fold change = +1.3), downregulation of CCNB1 (DFP 2x IC_{50} log2 fold change = -2.3, JIB-04 2x IC_{50} log2 fold change = -1.2), downregulation of AURKA (DFP 2x IC_{50} log2 fold change = -1.7, JIB-04 1μM log2 fold change = -0.7), and downregulation of AURKB (DFP 2x IC_{50} log2 fold change = -1.2, JIB-04 2x IC_{50} log2 fold change = -1.0). Additionally, JIB-04 2x IC_{50} upregulated PTEN, CDKN2D, and BCL10 (log2 fold change = +1.0, +1.5, +1.6, respectively) and downregulated CDK5 and EPHB4 (log2 fold change = -1.3, -1.0, respectively).

Table S1. Effects of DFP and JIB-04 on genes involved in JIB-04 KDM inhibition. The significance of log2 fold change DFP 2x IC_{50} and JIB-04 2x IC_{50} treatment p-values of DFP 2x IC_{50} and JIB-04 2x IC_{50} treatment relative to DMSO in MDA-MB-231 and MCF-7 cells. Gene names arranged in descending order of expression levels resulting from JIB-04 treatment to MDA-MB-231 cells. ns = not significant.

	**MDA-MB-231 (TNBC)**		**MCF-7 (ERα+ BCa)**	
GeneID	**DFP 2x IC_{50}**	**JIB-04 2x IC_{50}**	**DFP 2x IC_{50}**	**JIB-04 2x IC_{50}**
CDKN1A	6.3e-63	4.0e-88	1.7e-23	2.3e-26
CCNB1	2.8e-28	3.1e-37	8.4e-19	9.0e-09
AURKA	2.0e-19	2.3e-26	5.2e-14	6.9e-05
BCL10	ns	9.0e-03	ns	1.2e-05
CDKN2D	ns	3.3e-02	ns	1.8e-03
PDCD4	ns	ns	ns	ns
FBXW7	ns	ns	ns	ns
AKT1	ns	ns	ns	7.1e-05
TSC1	ns	ns	ns	ns
BCL2L11	ns	ns	ns	ns
CDCA7	2.5e-02	2.5e-03	ns	ns
AURKB	4.3e-02	5.4e-03	7.6e-05	7.7e-04
SIX1	ns	ns	ns	ns
RRAS	ns	ns	ns	4.7e-02
CASP8	ns	ns	ns	3.0e-02
PDGFRB	ns	ns	ns	ns
GLI1	ns	ns	ns	ns
FOXO4	ns	ns	ns	ns
CDK18	ns	ns	ns	ns
CDKN1C	ns	ns	ns	ns
APC	ns	ns	ns	ns
CDK5	ns	ns	ns	2.3e-04
EPHB4	ns	ns	ns	3.22e-05
PTEN	ns	ns	4.0e-02	6.0e-04
FOXM1	ns	ns	3.6e-02	ns

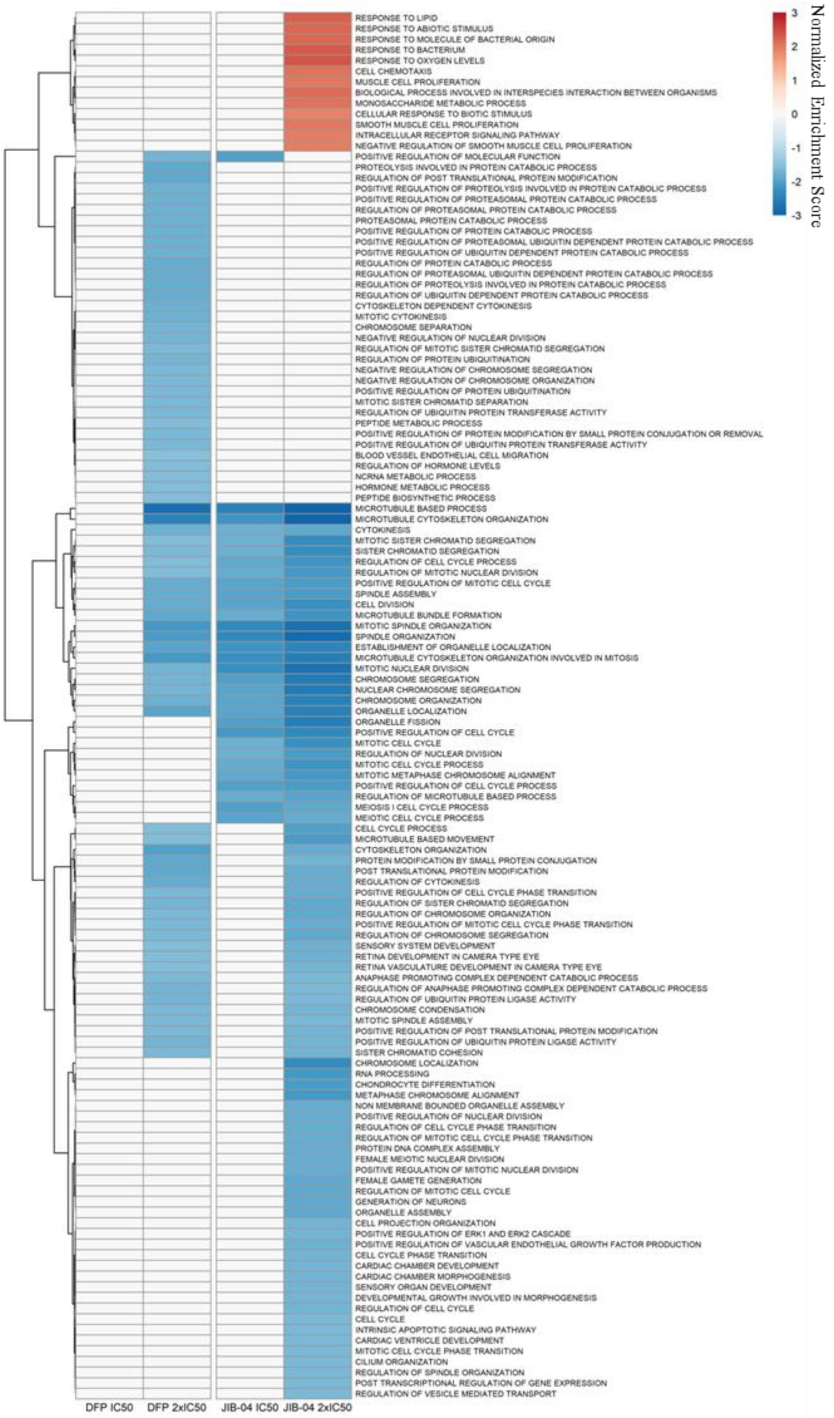

Figure S2. Full Gene Ontology Biological Processes (GOBP) Gene Set Enrichment Analysis (GSEA) heatmap of DFP and JIB-04 treatment of MDA-MB-231 cells. Significant gene set enrichment was defined by $p < 0.05$ and false discovery rate (FDR) < 0.25. DFP IC_{50} did not significantly enrich GOBP gene sets, DFP 2x IC_{50}, JIB-04 IC_{50}, and JIB-04 2x IC_{50} negatively enriched gene sets related to cell cycle processes, chromosomal arrangement, protein modification, cellular localization, and response to stimuli. Gene sets relating to cellular responses were positively enriched by JIB-04 2x IC_{50}.

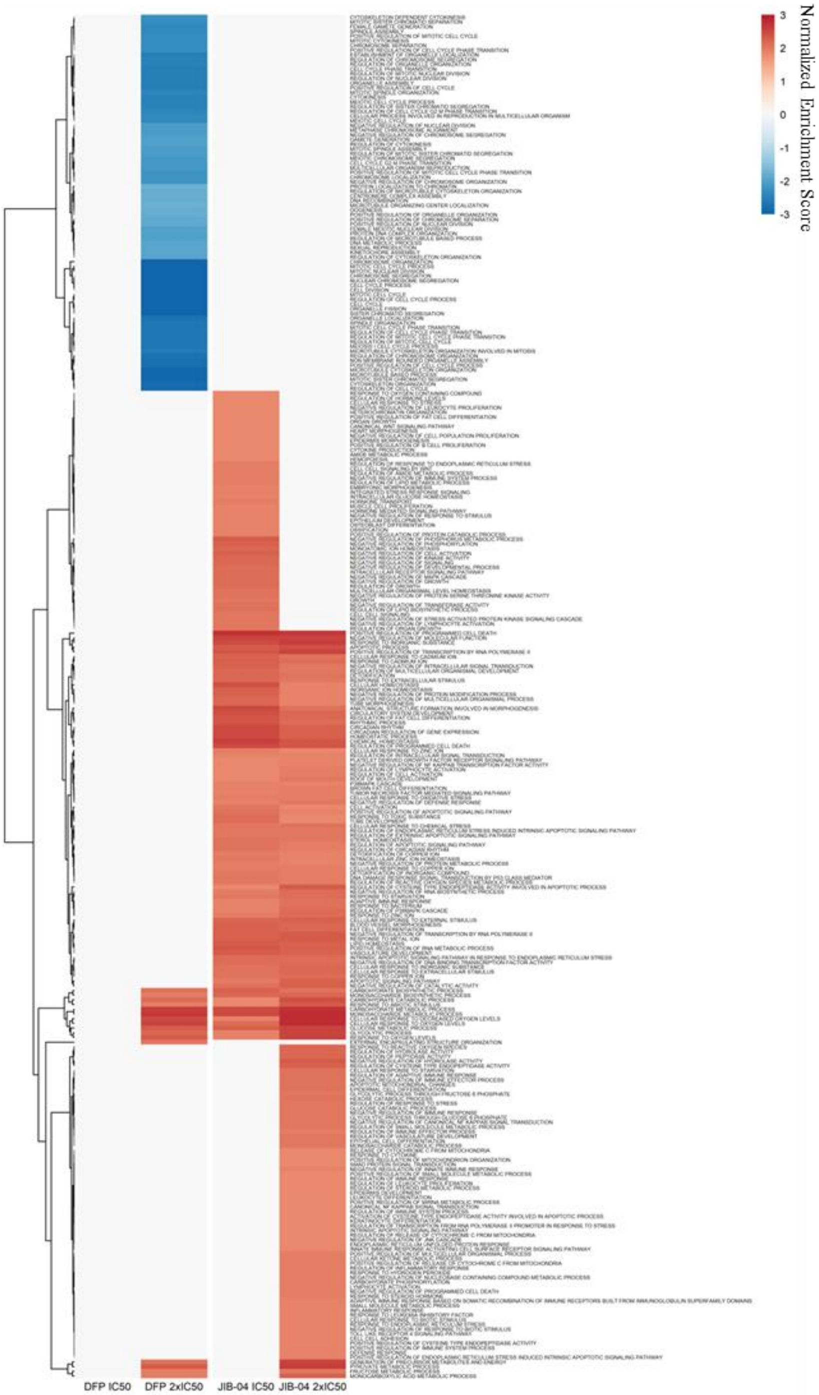

Figure S3. Full Gene Ontology Biological Processes (GOBP) Gene Set Enrichment Analysis (GSEA) heatmap of DFP and JIB-04 treatment to MCF-7 cells. Significant gene set enrichment was defined by $p < 0.01$ and false discovery rate (FDR) < 0.10. DFP IC_{50} did not significantly enrich GOBP gene sets. DFP 2x IC_{50}, JIB-04 IC_{50}, and JIB-04 2x IC_{50} positively enriched gene sets related to oxygen level response and metabolic processes. Individually, DFP 2x IC_{50} negatively enriched gene sets related to cell cycle processes and chromosomal arrangement, JIB-04 at IC_{50} and 2x IC_{50} positively enriched gene sets related to cellular responses to metal ions, apoptotic and tumor necrotic factor signaling pathways.

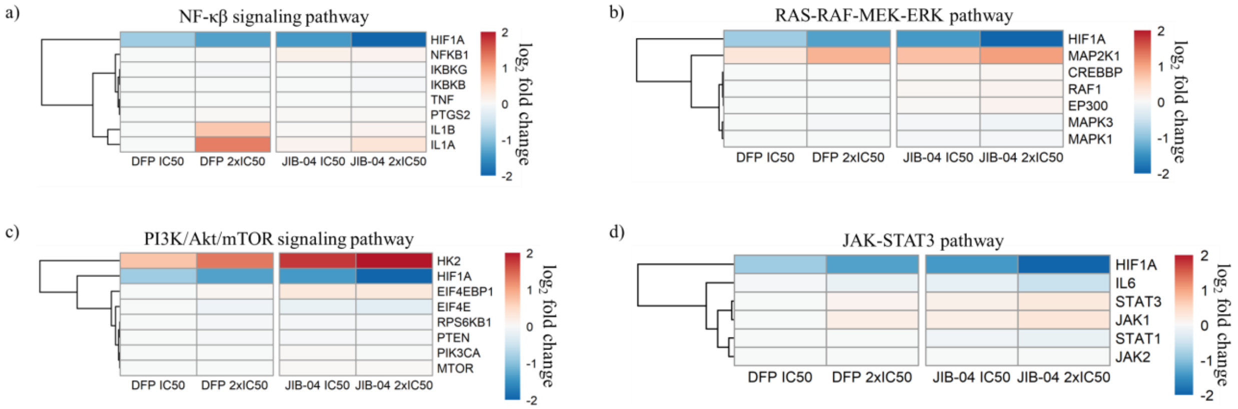

Figure S4. Effects of DFP and JIB-04 on traditional hypoxia inducible factor 1α (HIF-1α) regulation pathways were minimally affected by the downregulation of HIF-1α a) NF-κβ signaling pathway, b) RAS-RAF-MEK-ERK pathway, c) PI3K/Akt/mTOR signaling pathway, and d) JAK-STAT3 pathway.

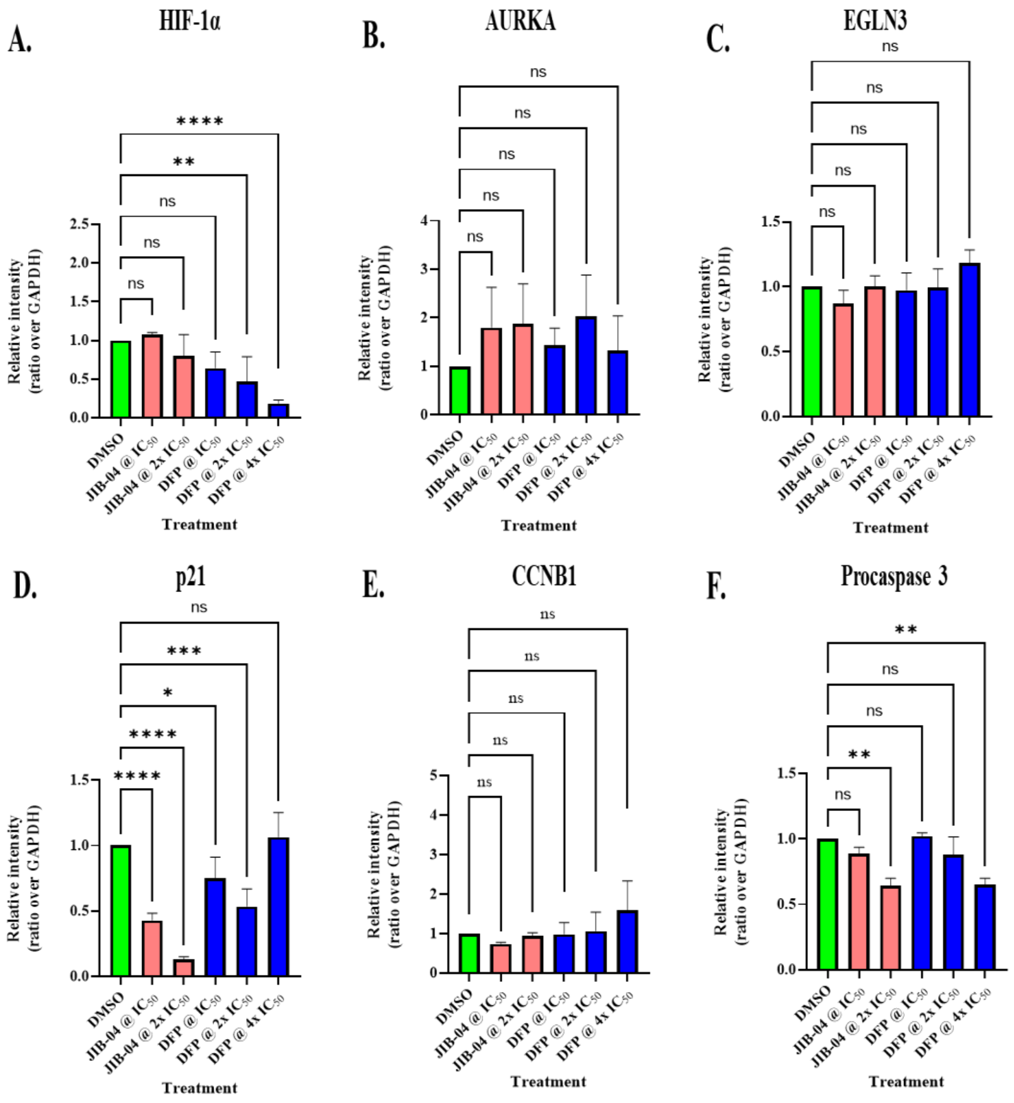

Figure S5. Western blot densitometric analyses (A-F) of MCF-7 cells treated with JIB-04 and DFP for 24 h. Quantification bars display means plus standard deviations; ordinary one-way ANOVA of each treatment was compared with the DMSO control group. *$p < 0.05$; **$p < 0.0048$; ***$p = 0.0001$; ****$p < 0.0001$.

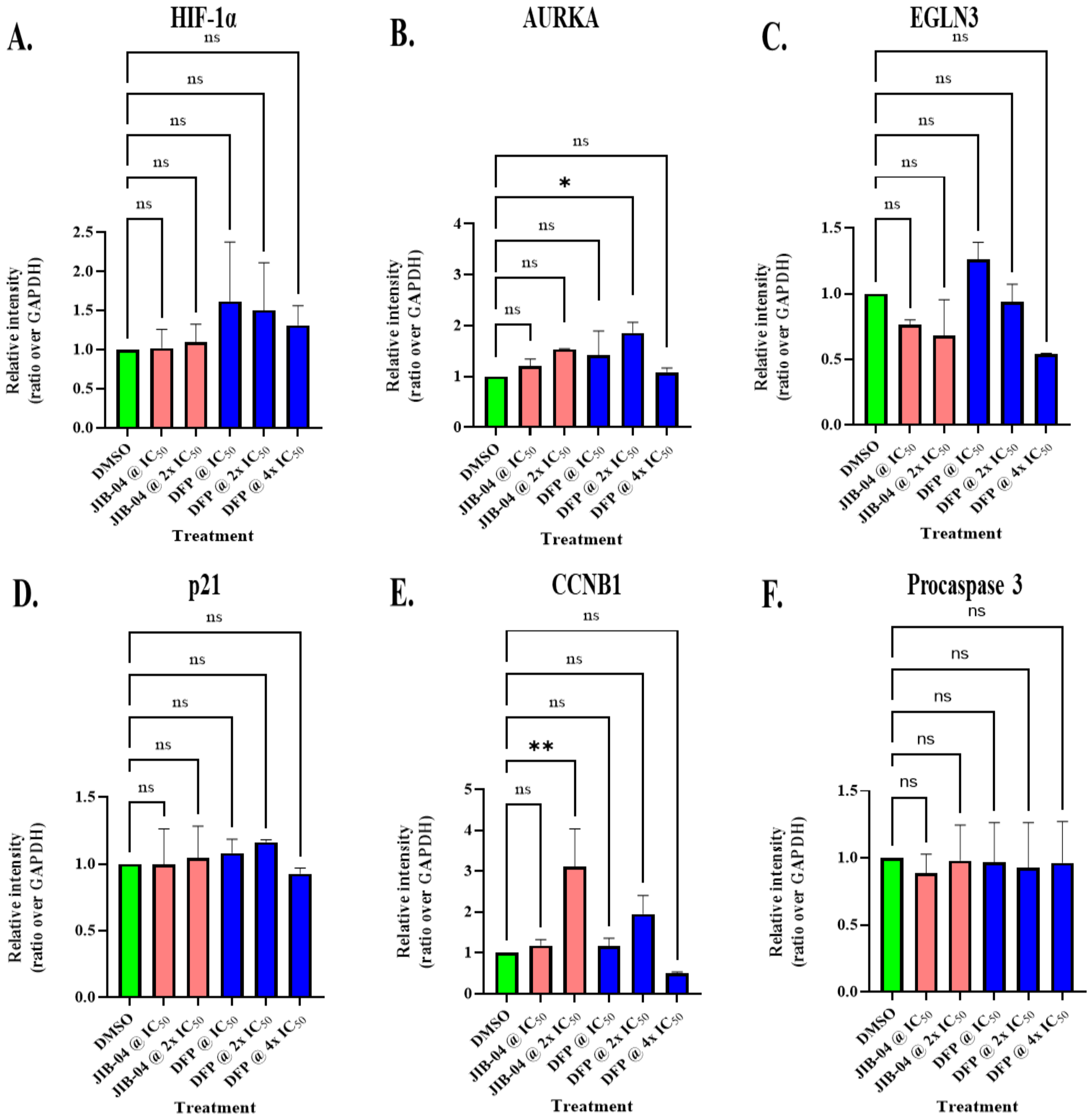

Figure S6. Western blot densitometric analyses (A-F) of MDA-MB-231 cells treated with JIB-04 and DFP for 24 h. Quantification bars display means plus standard deviations; ordinary one-way ANOVA of each treatment was compared with the DMSO control group. *$p < 0.05$; **$p < 0.001$.

Full Western blot gels with labels

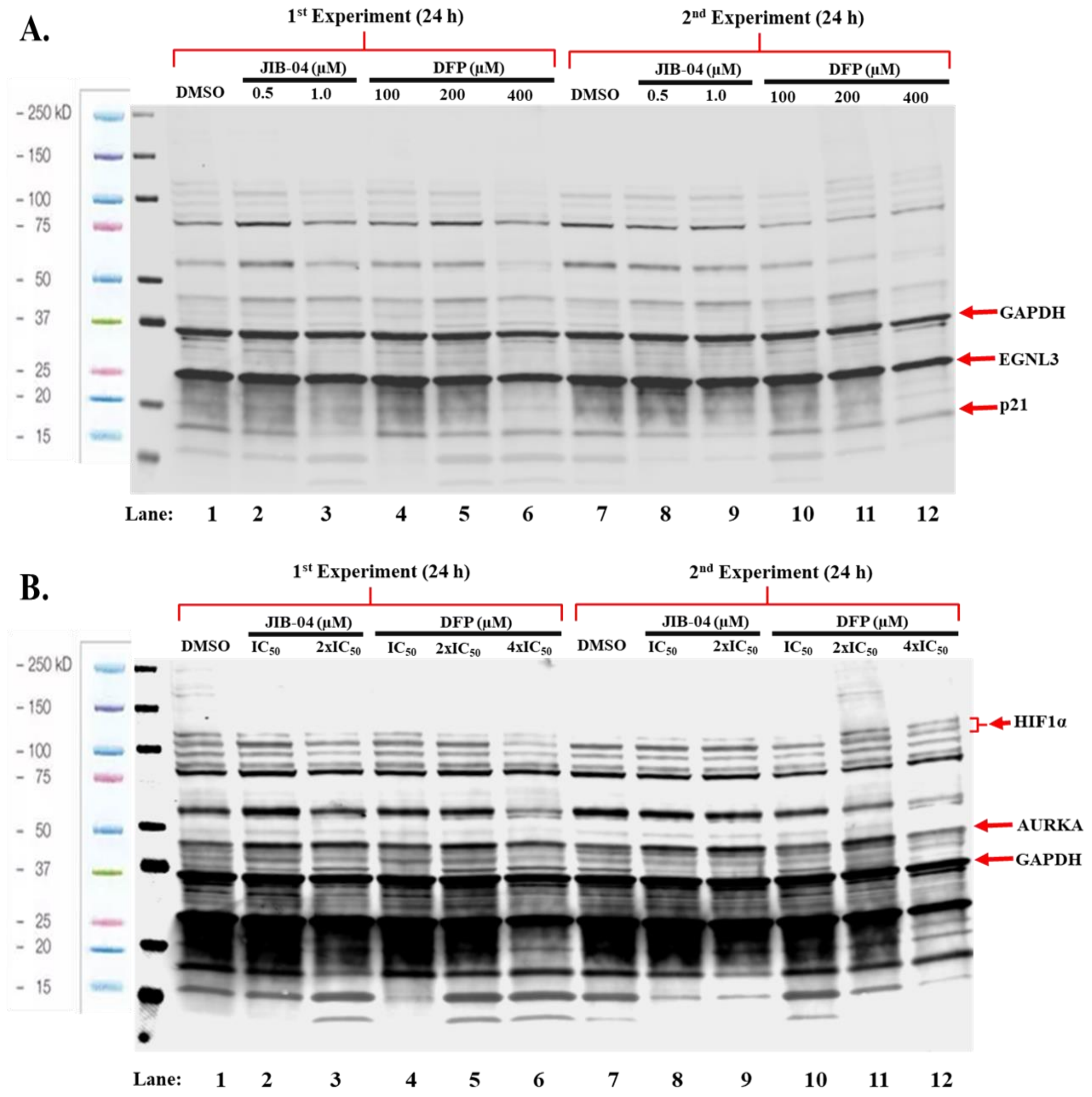

Figure S7. Full immunoblot gel images (A-B) of MCF-7 cells treated with JIB-04 and DFP for 24 h: *HIF1α antibody cocktail.*

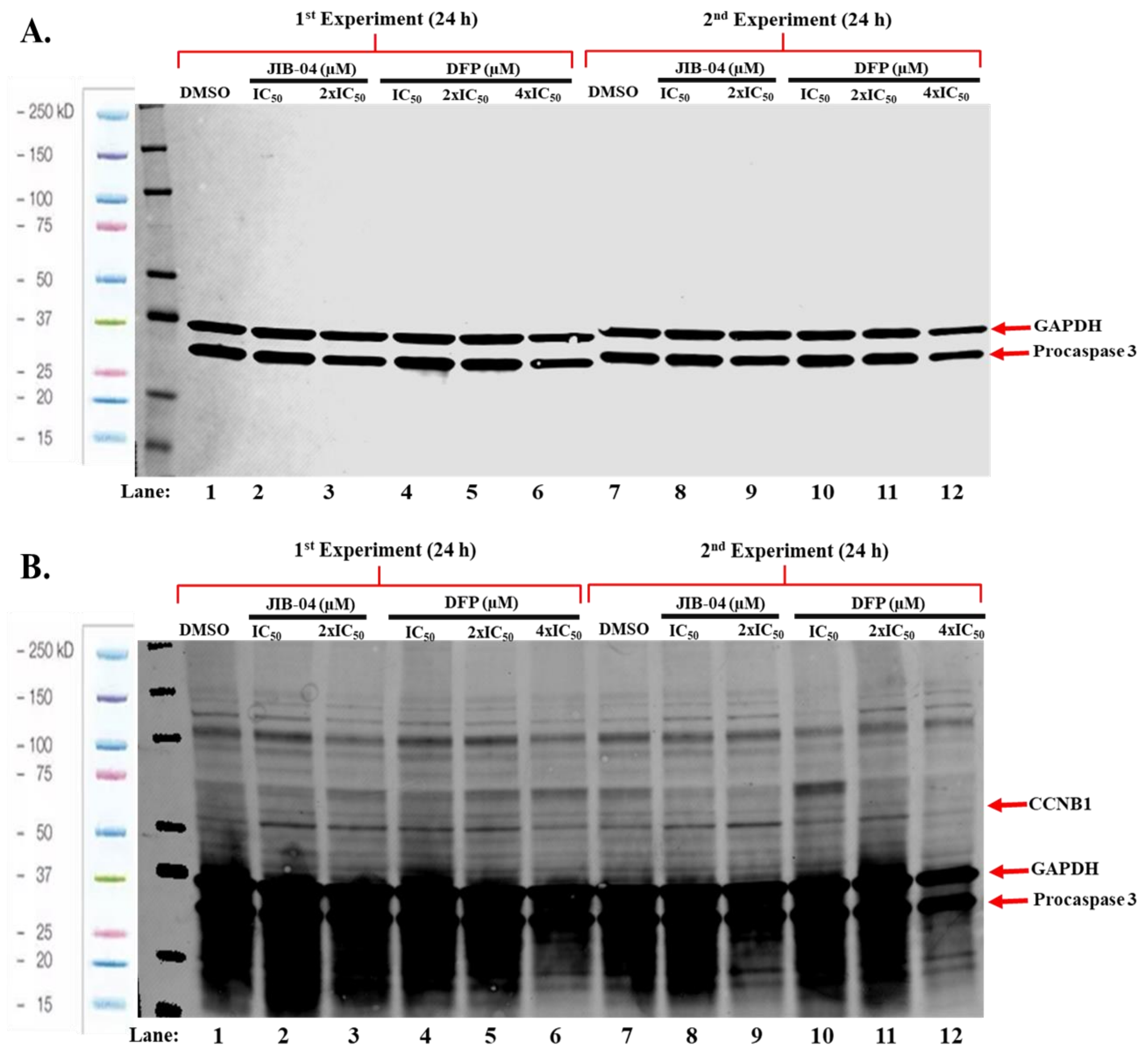

Figure S8. Full immunoblot gel images (A-B) of MCF-7 cells treated with JIB-04 and DFP for 24 h: *CCNB1 antibody cocktail.*

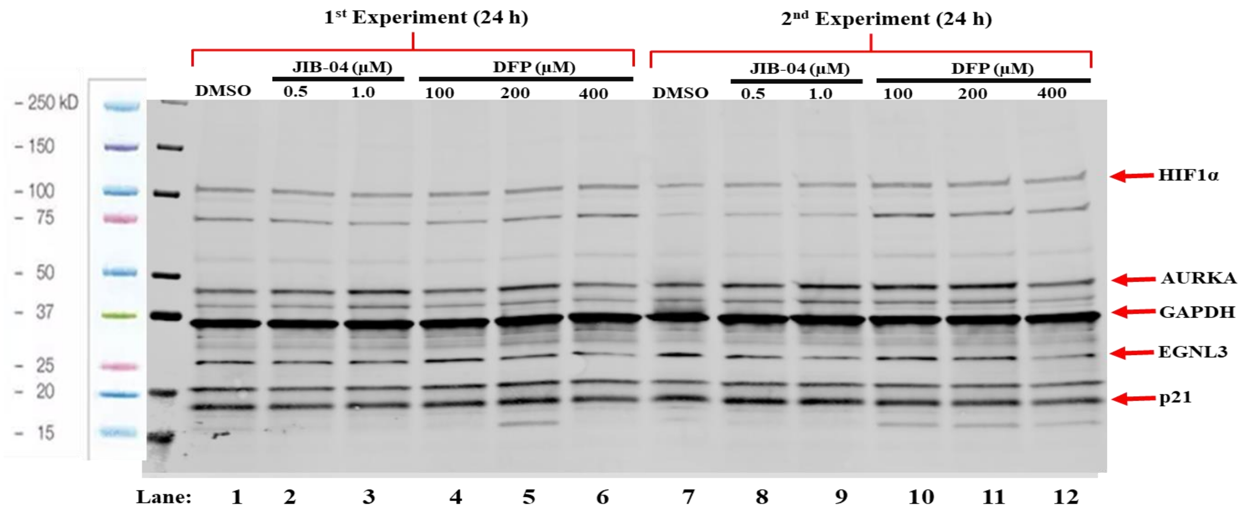

Figure S9. Full immunoblot gel images of MDA-MB-231 cells treated with JIB-04 and DFP for 24 h: *HIF1α antibody cocktail.*

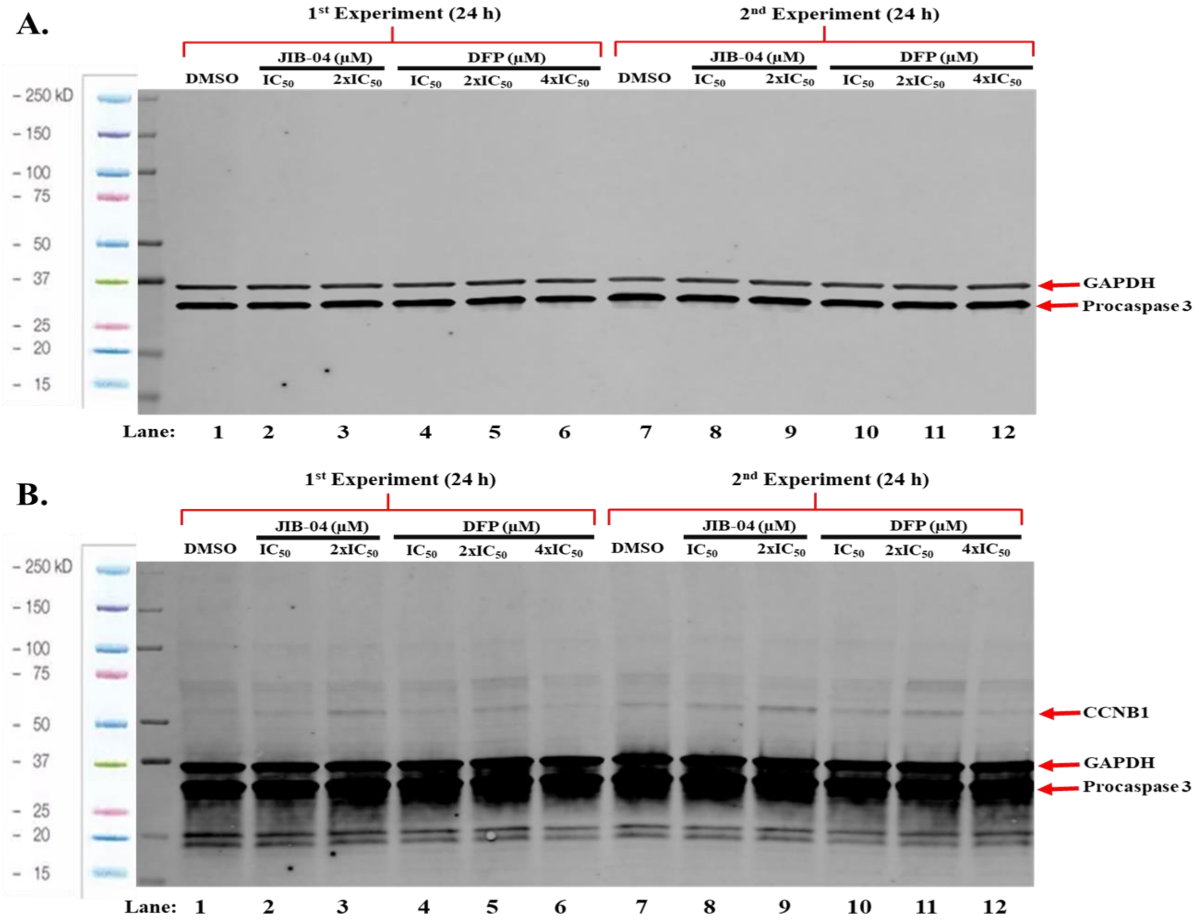

Figure S10. Full immunoblot gel images (A-B) of MDA-MB-231 cells treated with JIB-04 and DFP for 24 h: *CCNB1 antibody cocktail.*

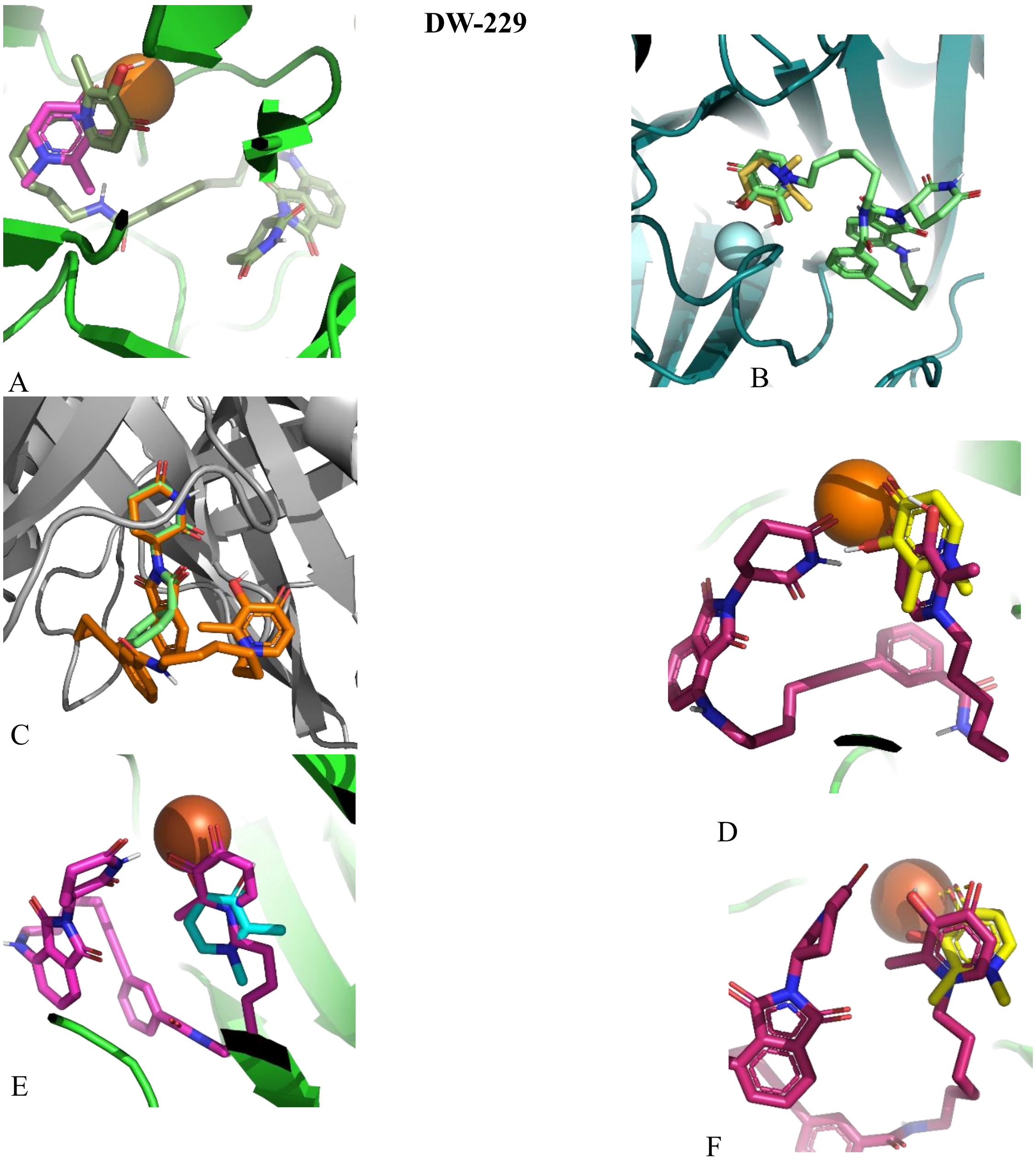

Figure S11. Molecular docking results of **DW-229** compared with DFP and thalidomide. (A) E3 Ligase (PDB: 4CI1) overlaying **DW-229** (orange, -9.507 kcal/mol) and thalidomide (green, -9.51 kcal/mol) show a large consistency in overlap between the active components of thalidomide. (B) KDM6A (PDB:3AVR) **DW-229** (green, -8.891 kcal/mol) DFP (yellow, -4.972 Kcal/mol) both show orientation towards the Fe (II) ion and are a plausible distance for chelation. (C) KDM5A (PDB:5CEH) **DW-229** (pink, -10.576 kcal/mol) and DFP (blue, -5.067 kcal/mol) demonstrate plausible coordination and potential chelation of the Fe (II) ion. (D) KDM5B (PDB:6H4Z) **DW-229** (Maroon, -10.821) and DFP (yellow-5.387 kcal/mol) both have their DFP moiety relatively close to the Fe (II) ion, but **DW-229** is flipped 180°. (E) KDM3A (PDB:2Q8C) **DW-229** (maroon, -10.644 kcal/mol) and DFP (yellow, -5.568 kcal/mol) both have the DFP moiety plausibly close

to the Fe (II) ion for chelation. (F) KDM2A (PDB:4QXB) **DW-229** (green, -9.479 kcal/mol) and DFP (pink, -5.435 kcal/mol), the position of **DW-229** DFP moiety seems not be well-oriented to optimally chelate the Fe (II) ion.

DW-449

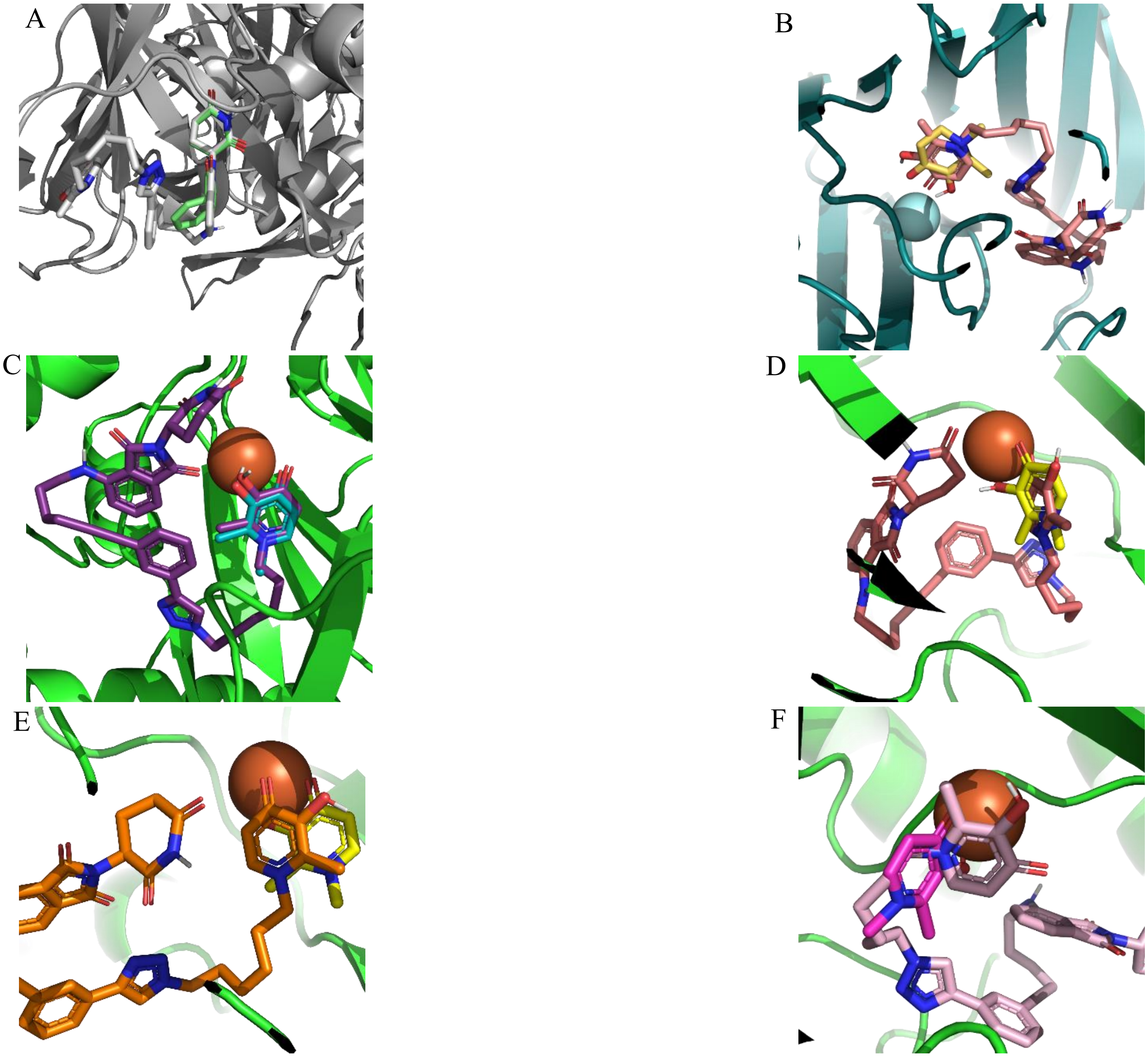

Figure S12. Molecular docking results of **DW-449** compared with DFP and thalidomide. (A) E3 Ligase (PDB: 4CI1) overlaying **DW-449** (white, -9.438 kcal/mol) and thalidomide (green, -9.51 kcal/mol) show a large consistency in overlap between the active components of thalidomide. **(**B) KDM6A (PDB:3AVR) **DW-449** (salmon, -9.773 kcal/mol) and DFP (yellow, -4.972 Kcal/mol) both show strong orientation towards the iron ion and are a plausible distance for chelation. (C) KDM5A (PDB:5CEH) **DW-449** (purple, -10.498 kcal/mol) and DFP (teal, -5.067 kcal/mol), the

docked output shows a large degree of overlap between DFP and the corresponding portion of **DW-449**, with plausible chelation. (D) KDM5B (PDB:6H4Z) docked output of **DW-449 (**salmon, -10.707 kcal/mol) and DFP (yellow, -5.387 kcal/mol) shows that their iron-binding groups are relatively close to the Fe (II) ion, although **DW-449** is not oriented for optimal chelation. (E) KDM3A (PDB:2Q8C) **DW-449** (orange, -9.859 kcal/mol) and DFP (yellow, -5.568 kcal/mol) both adopt orientations showing their iron-binding groups are plausibly close to the Fe (II) ion but not oriented for optimal chelation. (F) KDM2A (PDB:4QXB) **DW-449 (**light pink, -10.768 kcal/mol) and DFP (dark pink, -5.435 kcal/mol) both adopt orientations showing their iron-binding groups are plausibly close to the Fe (II) ion but not oriented for optimal chelation.

DW-451

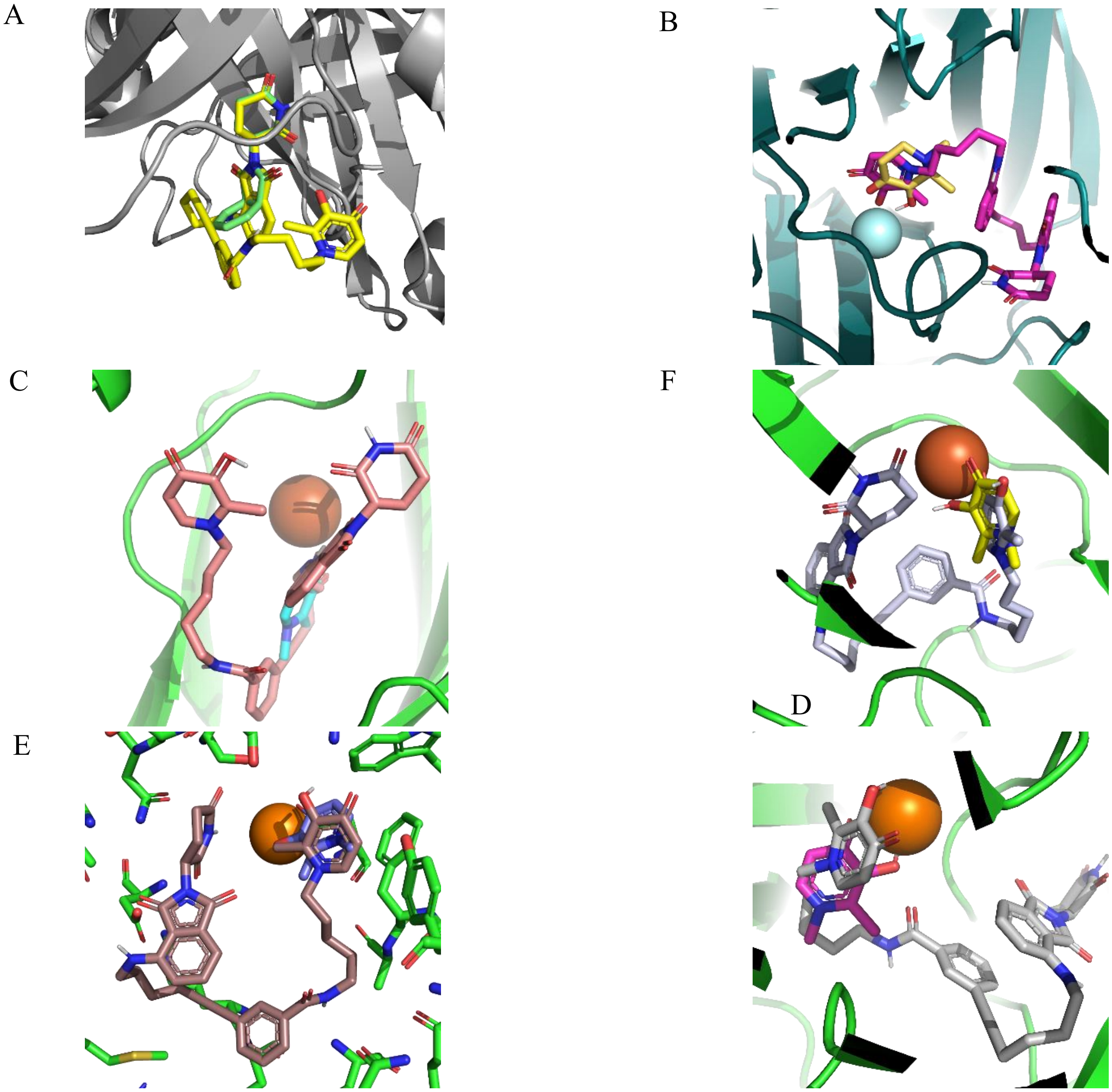

Figure S13. Molecular docking results of **DW-451** compared with DFP and thalidomide. (A) E3 Ligase (PDB: 4CI1) overlaying **DW-451** (yellow, -10.026 kcal/mol) and thalidomide (green, -9.51 kcal/mol) show a large consistency in overlap between the active components of thalidomide. (B)

KDM6A (PDB:3AVR) **DW-451** (pink, -8.143 kcal/mol) and DFP (yellow, -4.972 kcal/mol) both show strong orientation towards the Fe (II) ion and are at a plausible distance for chelation. **(**C) KDM5A (PDB:5CEH) **DW-451** (salmon, -10.945 kcal/mol) and DFP (blue, -5.067 kcal/mol) adopt docked orientations showing that their iron-binding groups are in proximity for plausible chelation with the Fe (II) ion. However, these orientations may not afford optimal chelation. **(**D) KDM5B (PDB:6H4Z) **DW-451** (gray, -11.186 kcal/mol) and DFP (yellow, -5.387 kcal/mol) adopt docked orientations showing that their iron-binding groups are in proximity for plausible chelation with the Fe (II) ion. However, **DW-451** is flipped from chelation. (E) KDM3A (PDB:2Q8C) **DW-451** (brown, -10.379 kcal/mol) and DFP (blue, -5.568 kcal/mol) the iron-binding group of **DW-451** is close to the Fe (II) ion, but not optimally oriented for chelation. (F) KDM2A (PDB:4QXB) **DW-451** (grey, -9.765 kcal/mol) and DFP (pink, -5.435 kcal/mol) the iron-binding group of **DW-451** is close to the Fe (II) ion, but not optimally oriented for chelation.

DW-455

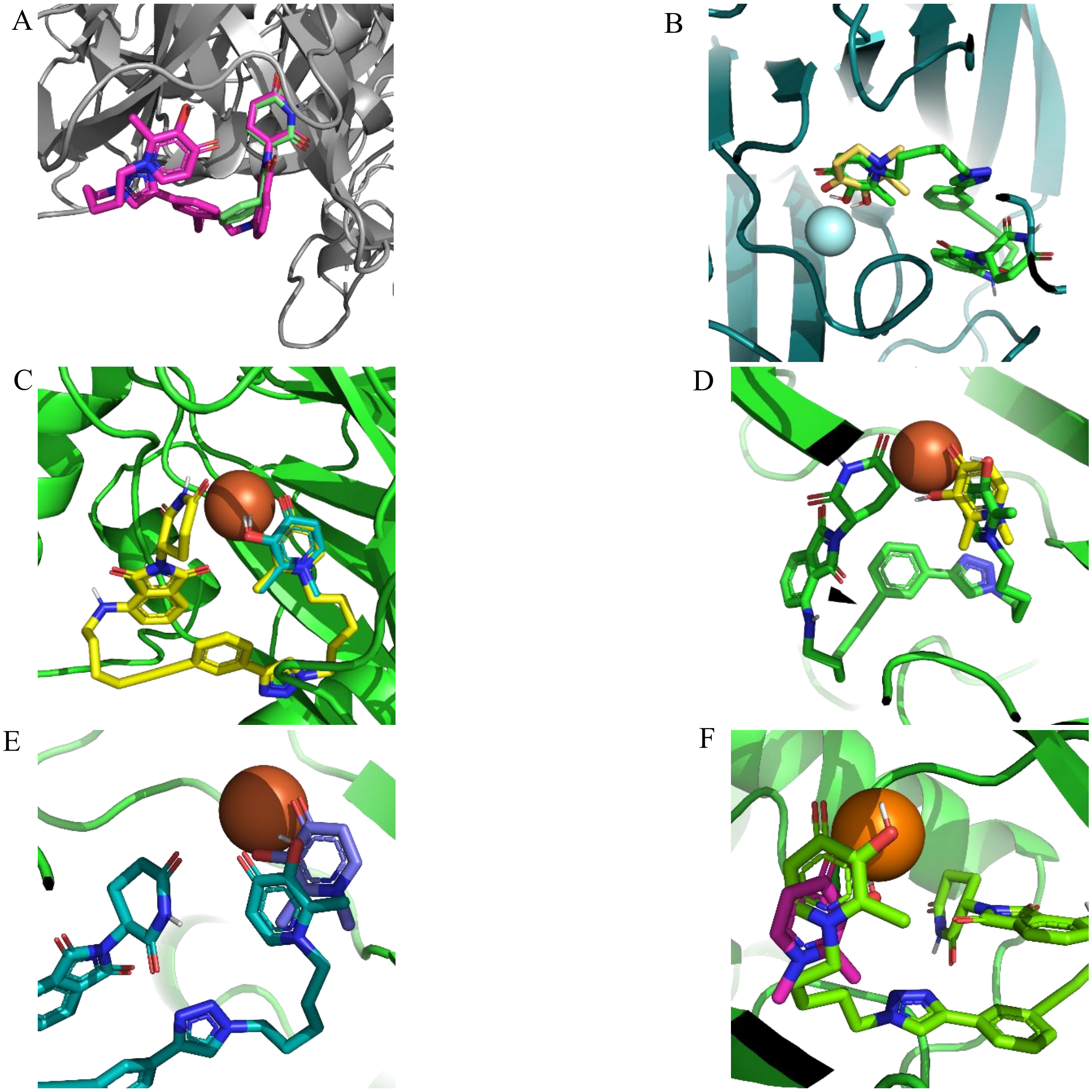

Figure S14. Molecular docking results of **DW-455** compared with DFP and thalidomide. (A) E3 Ligase (PDB: 4CI1) overlaying **DW-455** (pink, -10.675 kcal/mol) and thalidomide (green, -9.51 kcal/mol) show a large consistency in overlap between the active components of thalidomide. (B) KDM6A (PDB:3AVR) **DW-455** (green, -9.018 kcal/mol) and DFP (yellow, -4.972 kcal/mol) both show strong orientation towards the Fe (II) ion and are at a plausible distance for chelation. (C) KDM5A (PDB:5CEH) **DW-455** (yellow, -10.11 kcal/mol) and DFP (blue, -5.067 kcal/mol) the docked output shows a large degree of overlap between DFP and the corresponding portion of **DW-455**. (D) KDM5B (PDB:6H4Z) **DW-455** (green, -10.112 kcal/mol) and DFP (yellow, -5.387 kcal/mol) both adopt docking poses such that their iron binding groups are oriented toward the Fe (II) ion, but **DW-455** is not oriented for optimal chelation. (E) KDM3A (PDB:2Q8C) **DW-455** (teal, -10.395 kcal/mol) and DFP (blue, -5.568 kcal/mol) iron binding groups are oriented toward the Fe (II) ion and are within a distance for chelation. (F) KDM2A (PDB:4QXB) **DW-455** (green, -9.665 kcal/mol) and DFP (pink, -5.435 kcal/mol) position their iron binding groups close to the Fe (II) ion. However, **DW-455** iron binding group is not oriented for optimal chelation.

Table S2. Docking scores of the designed CRBN E3 ligase DFP-based PROTACs.

**Protein (PDB)**	**Docking Scores Kcal/mol)**					
	CRBN	DFP	**DW-229**	**DW-449**	**DW-451**	**DW-455**
CRBN (4CI1)	-9.51		-9.507	-9.438	-10.026	-10.675
KDM6A (3AVR)		-4.972	-8.891	-9.773	8.143	-9.018
KDM5A (5CEH)		-5.067	-10.576	-10.498	-10.945	-10.11
KDM5B (6H4Z)		-5.387	-10.821	-10.707	-11.186	-10.112
KDM3A (2Q8C)		-5.568	-10.664	-9.859	-10.379	-10.395
KDM2A (4QXB)		5.435	-9.479	-10.768	-9.765	-9.665

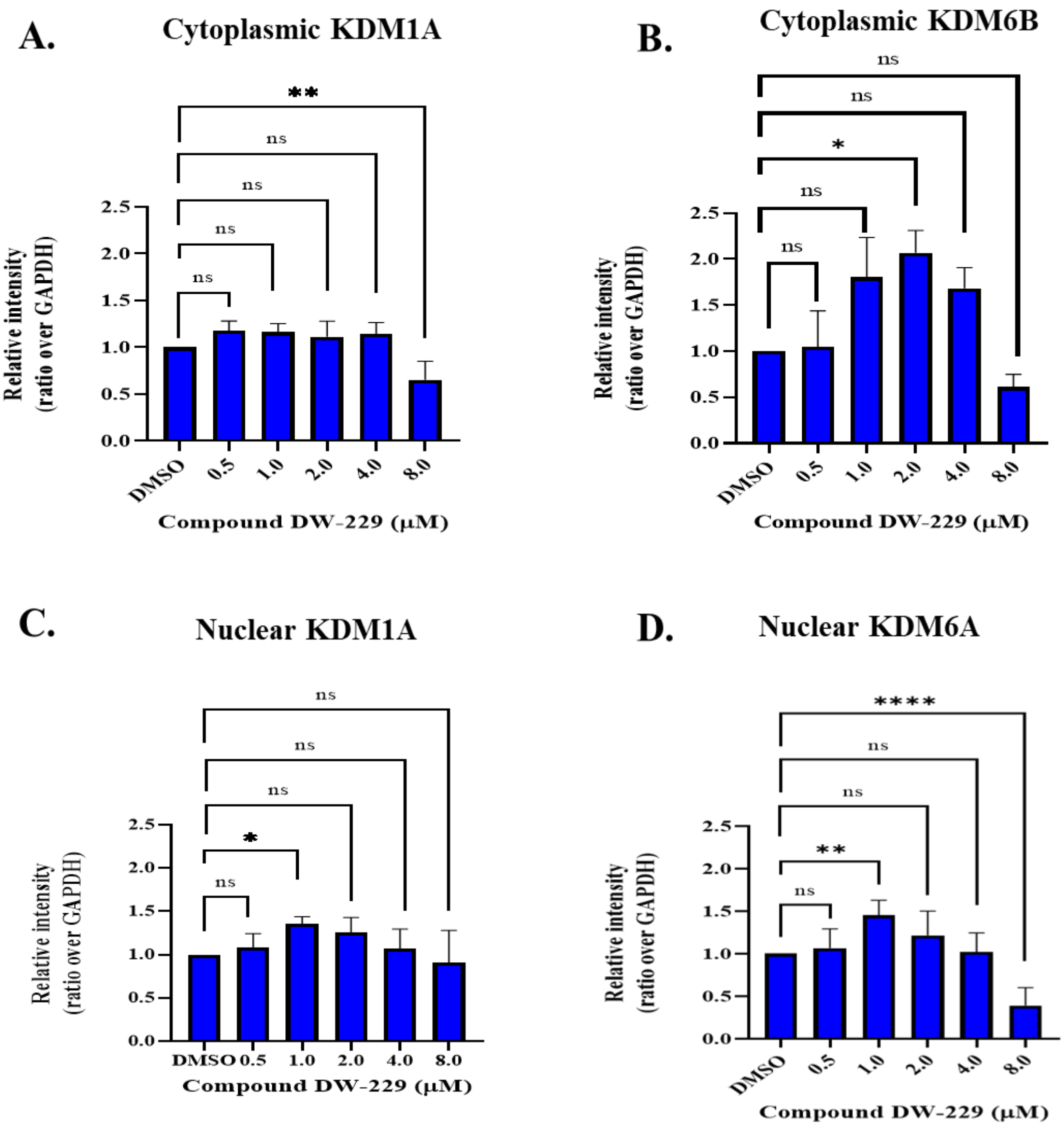

Figure S15. Western blot densitometric analyses (A–D) of MCF-7 cells treated with **DW-229** for 24 h: *KDM1A, KDM6A, and KDM6B antibody cocktail.* Quantification bars display means plus standard deviations of data obtained from at least two independent experiments; ordinary one-way ANOVA of each treatment was compared with the DMSO control group. *p < 0.05; **p < 0.005; ***p = 0.001; ****p < 0.0001).

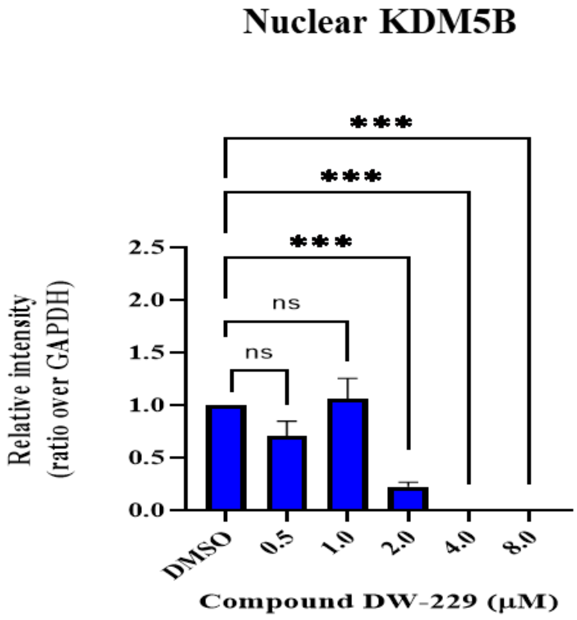

Figure S16. Western blot densitometric analysis of MCF-7 cells treated with **DW-229** for 24 h: *KDM5B antibody cocktail.* Quantification bars display means plus standard deviations of data obtained from at least two independent experiments; ordinary one-way ANOVA of each treatment was compared with the DMSO control group. ***p = 0.001.

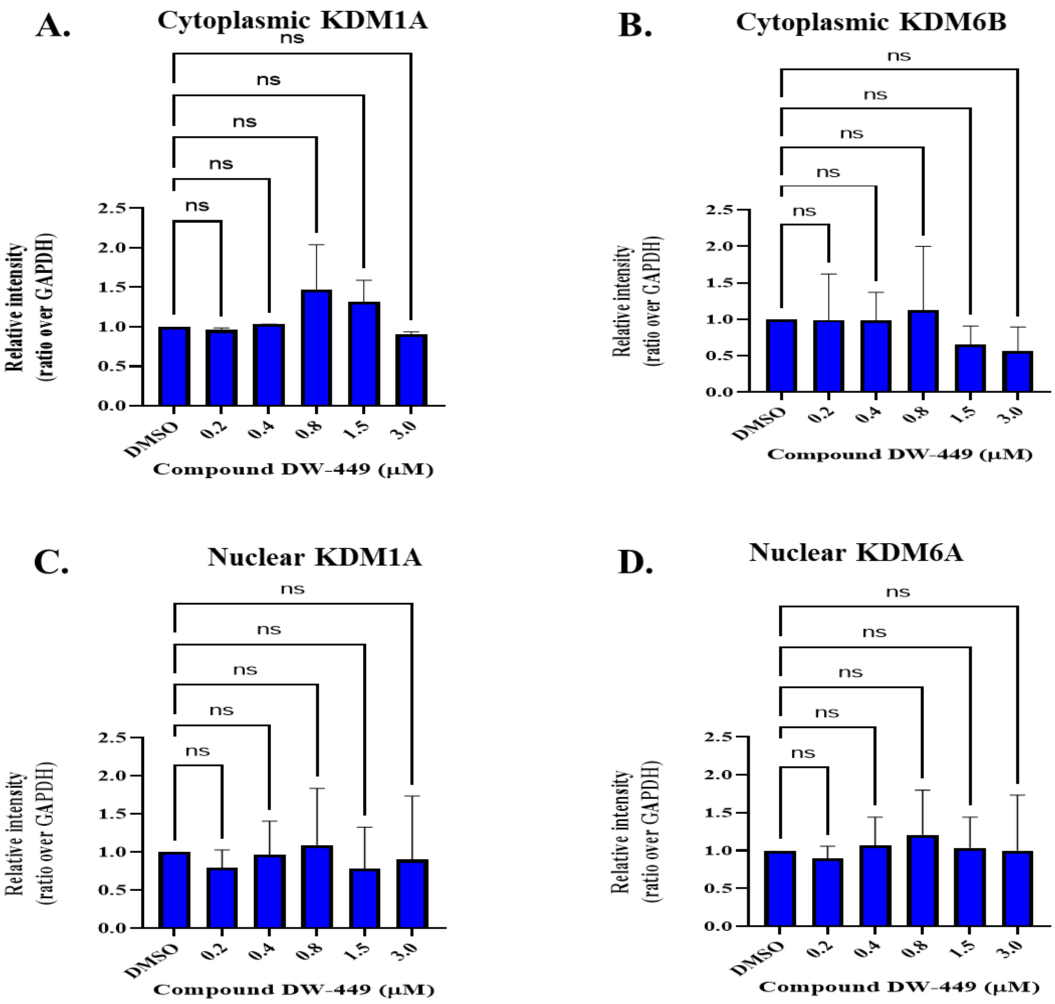

Figure S17. Western blot densitometric analyses (A–D) of MCF-7 cells treated with **DW-449** for 24 h: *KDM1A, KDM6A, and KDM6B antibody cocktail.* Quantification bars display means plus standard deviations of data obtained from at least two independent experiments; ordinary one-way ANOVA of each treatment was compared with the DMSO control group.

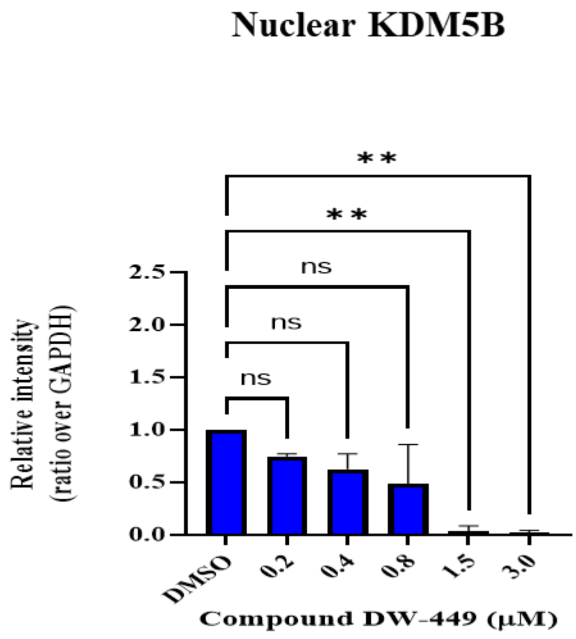

Figure S18. Western blot densitometric analysis of MCF-7 cells treated with **DW-449** for 24 h: *KDM5B antibody cocktail.* Quantification bars display means plus standard deviations of data obtained from at least two independent experiments; ordinary one-way ANOVA of each treatment was compared with the DMSO control group. $^{**}p < 0.005$.

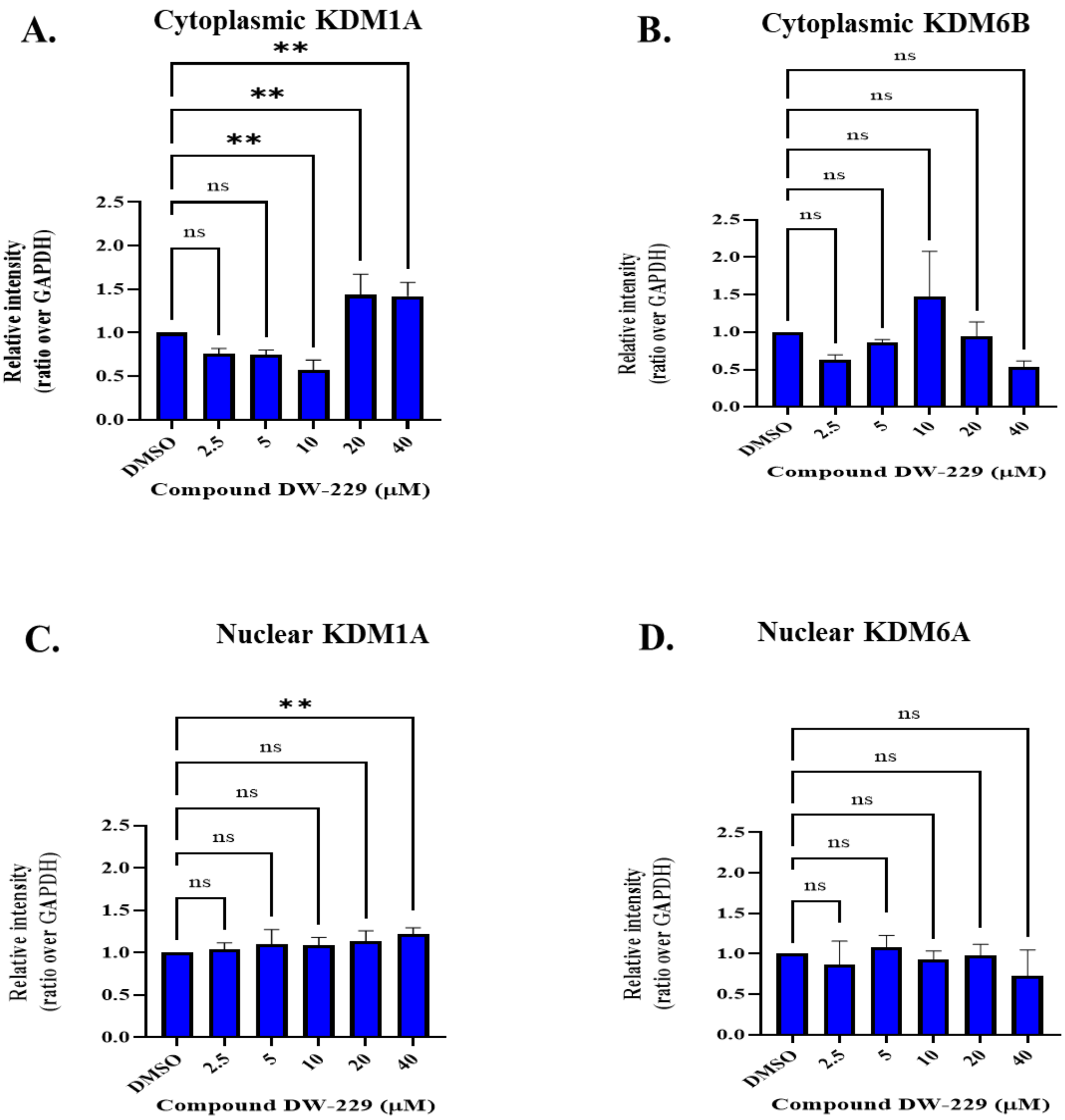

Figure 19. Western blot densitometric analyses (A–D) of MDA-MB-231 cells treated with **DW-229** for 24 h: *KDM1A, KDM6A, and KDM6B antibody cocktail.* Quantification bars display means plus standard deviations of data obtained from at least two independent experiments; ordinary one-way ANOVA of each treatment was compared with the DMSO control group. $^{**}p < 0.005$.

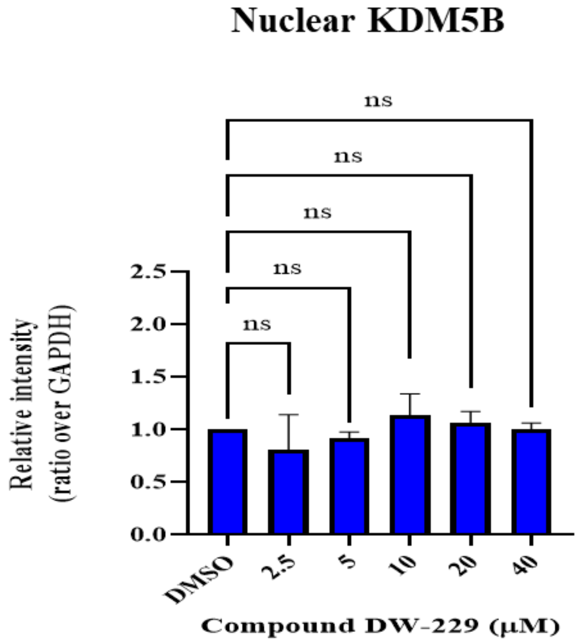

Figure 20. Western blot densitometric analysis of MDA-MB-231 cells treated with **DW-229** for 24 h: *KDM5B antibody cocktail.* Quantification bars display means plus standard deviations of data obtained from at least two independent experiments; ordinary one-way ANOVA of each treatment was compared with the DMSO control group.

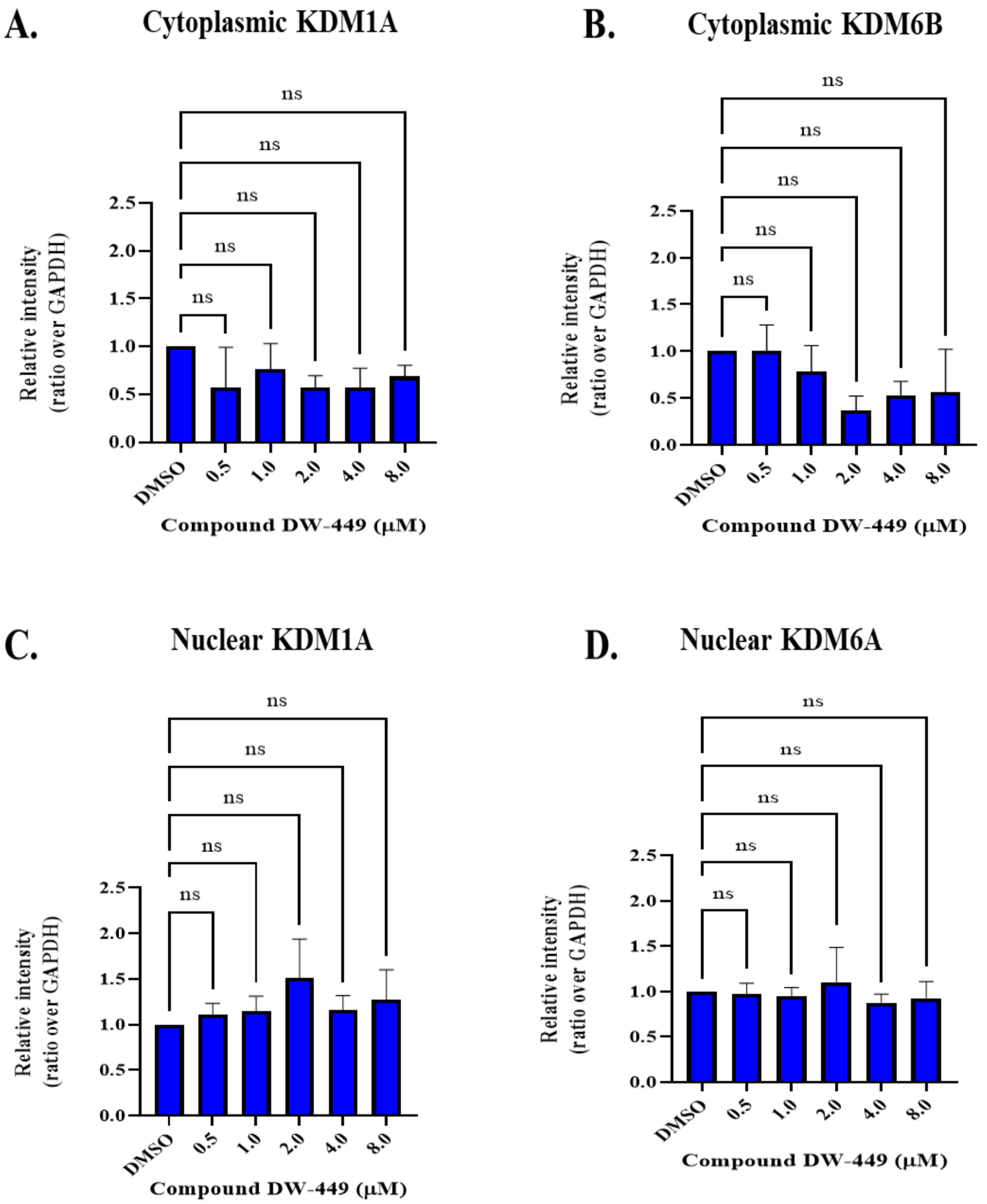

Figure 21. western blot densitometric analysis (A–D) of MDA-MB-231 cells treated with **DW-449** for 24 h: *KDM1A, KDM6A, and KDM6B antibody cocktail.* Quantification bars display means plus standard deviations of data obtained from at least two independent experiments; ordinary one-way ANOVA of each treatment was compared with the DMSO control group.

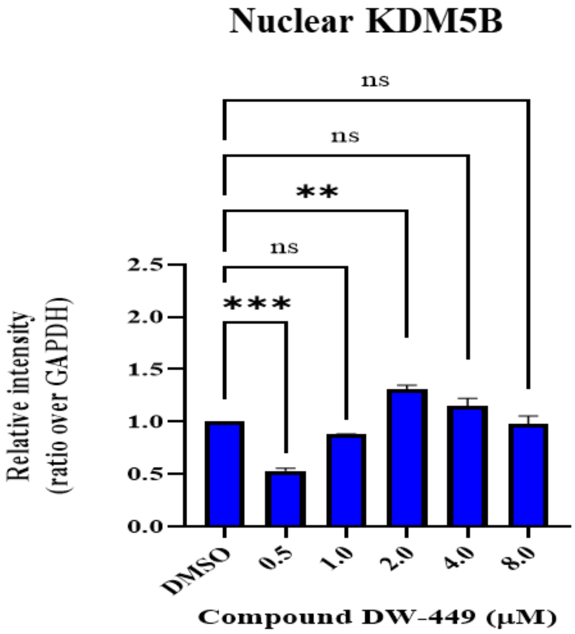

Figure 22. Western blot densitometric analysis of MDA-MB-231 cells treated with **DW-449** for 24 h: *KDM5B antibody cocktail.* Quantification bars display means plus standard deviations of data obtained from at least two independent experiments; ordinary one-way ANOVA of each treatment was compared with the DMSO control group. $^{**}p < 0.005$; $^{***}p = 0.001$.

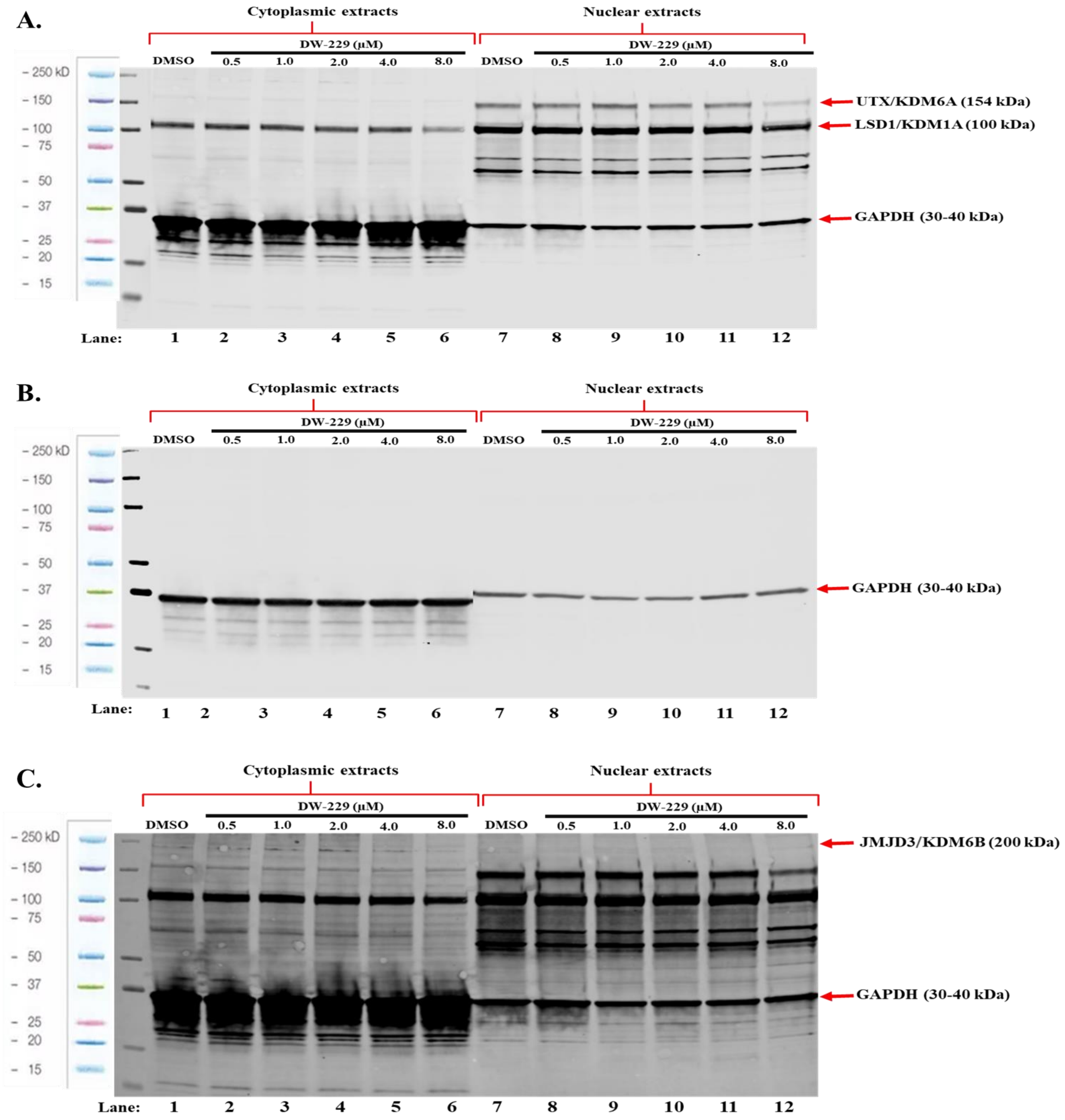

Figure S23. Full immunoblot gel images (A-C) of MCF-7 cells treated with **DW-229** for 24 h: *KDM1A, KDM6A, and KDM6B antibody cocktail.*

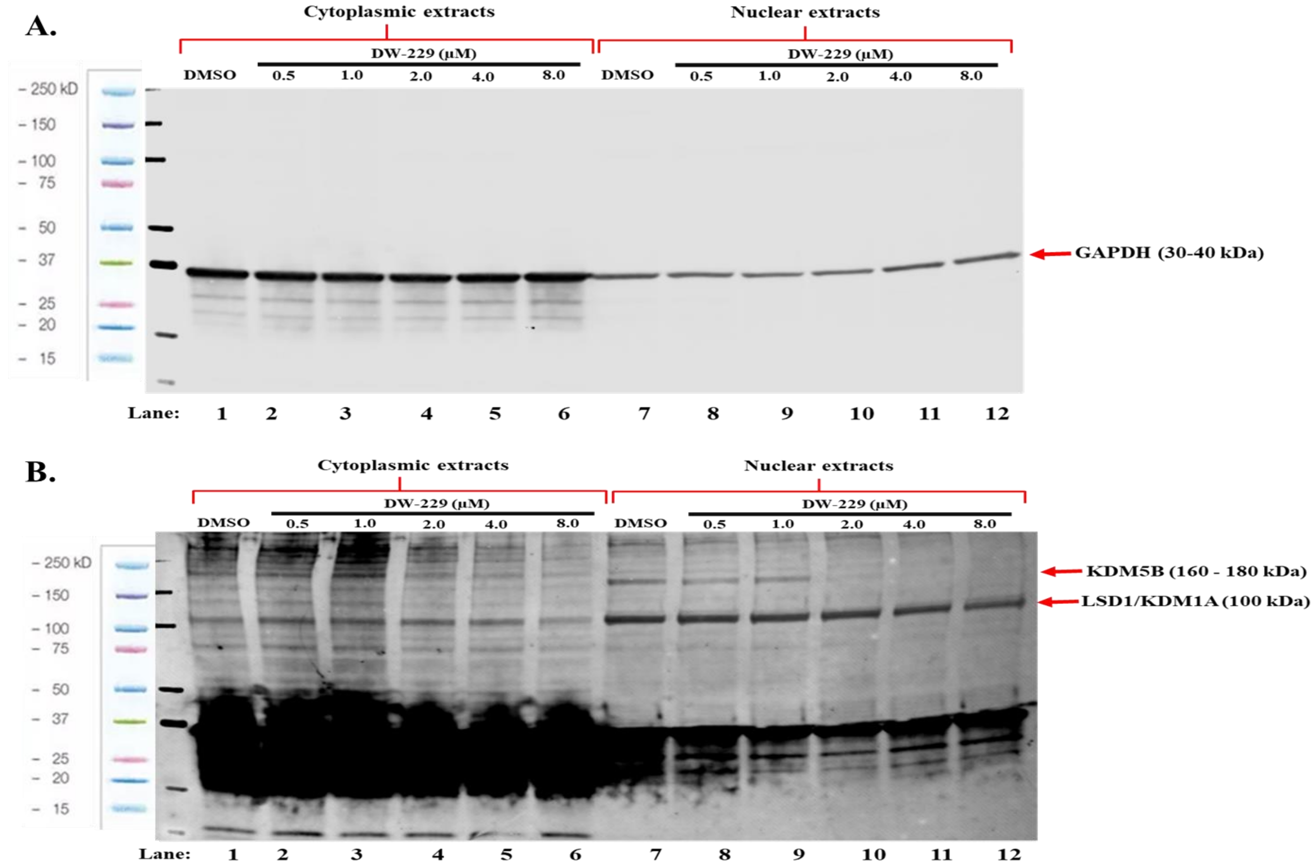

Figure S24. Full immunoblot gel images (A-B) of MCF-7 cells treated with **DW-229** for 24 h: *KDM5B antibody cocktail.*

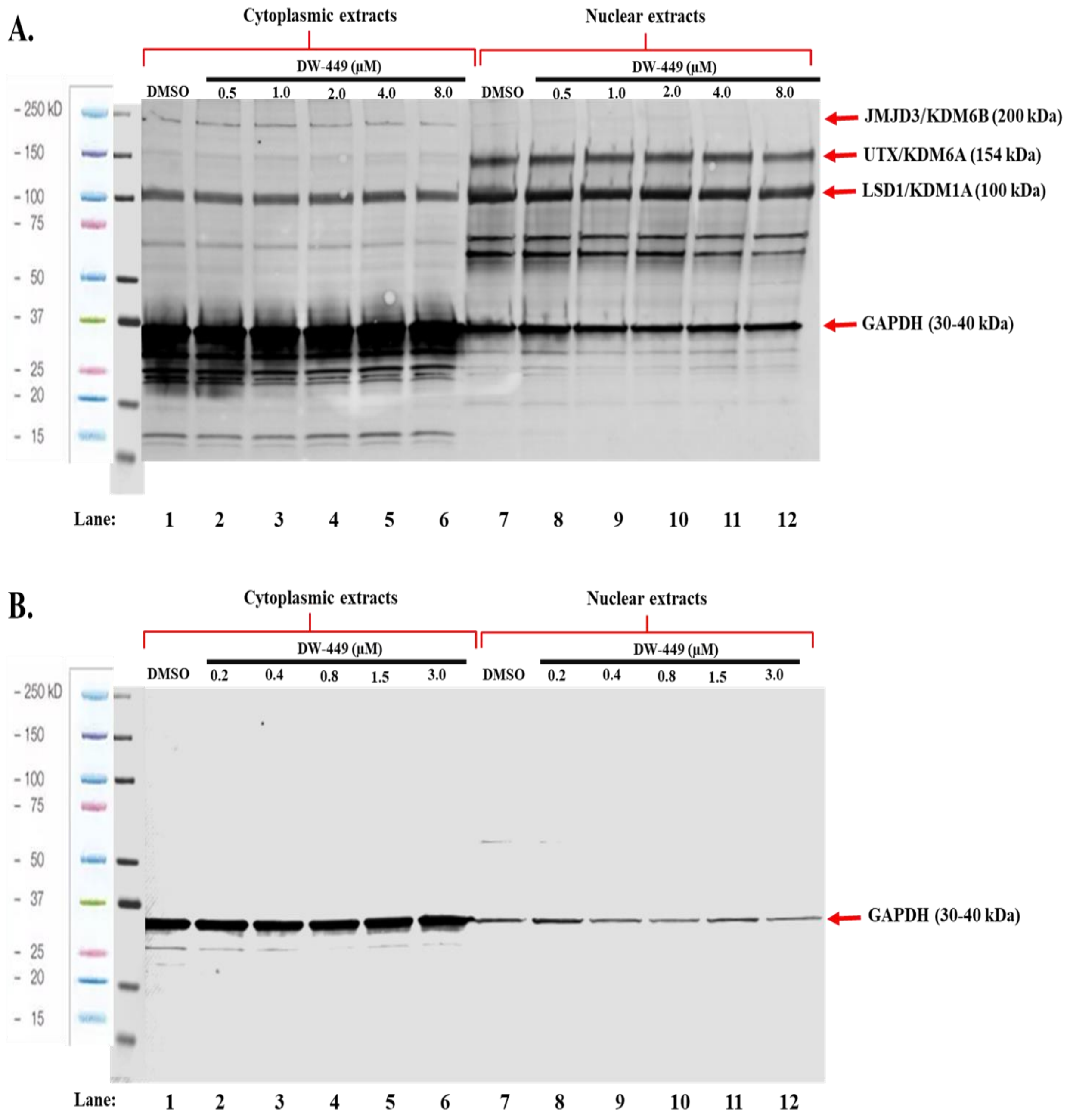

Figure S25. Full immunoblot gel images (A-B) of MCF-7 cells treated with **DW-449** for 24 h: *KDM1A, KDM6A, and KDM6B antibody cocktail.*

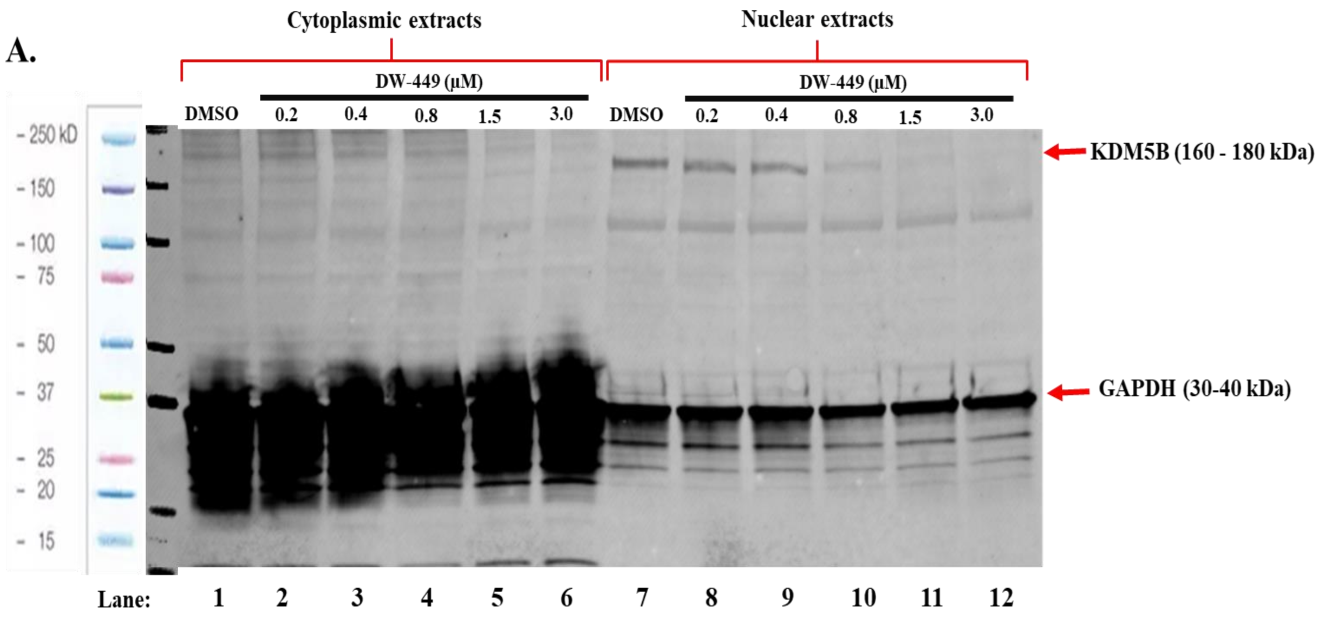

Figure S26. Full immunoblot gel images (A-B) of MCF-7 cells treated with **DW-449** for 24 h: *KDM5B antibody cocktail.*

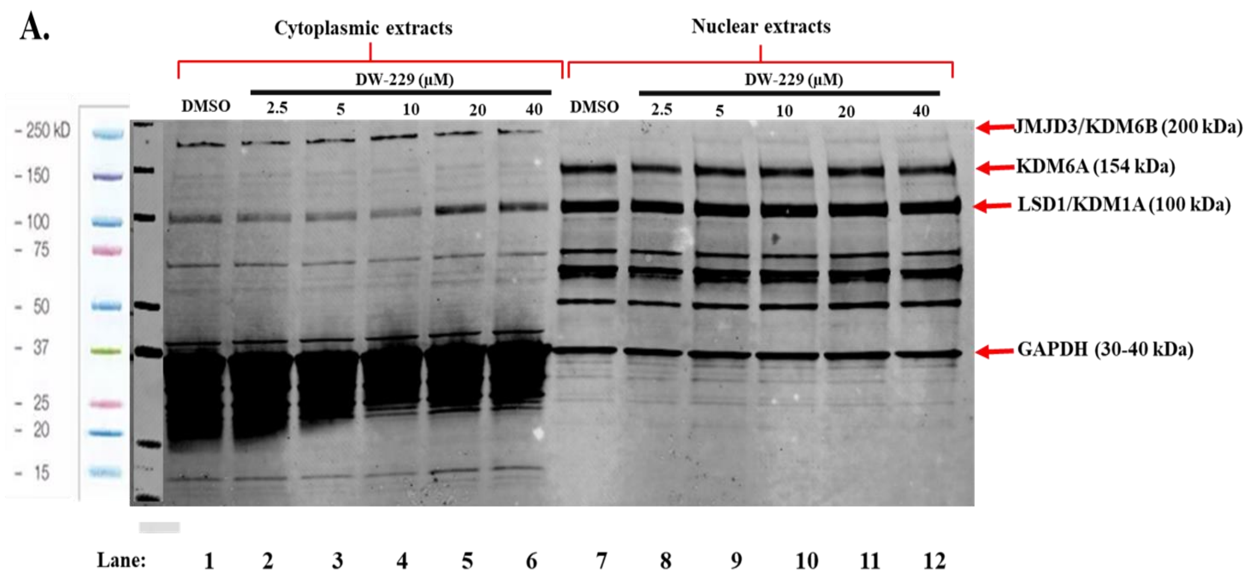

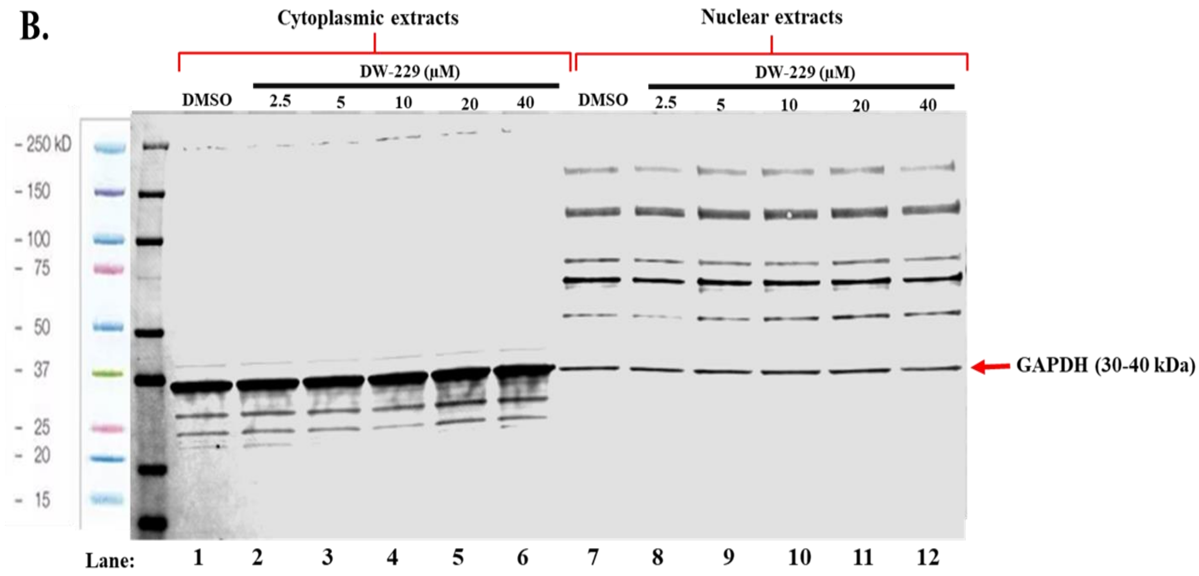

Figure S27. Full immunoblot gel images (A-B) of MDA-MB-231 cells treated with **DW-229** for 24 h: *KDM1A, KDM6A, and KDM6B antibody cocktail.*

A.

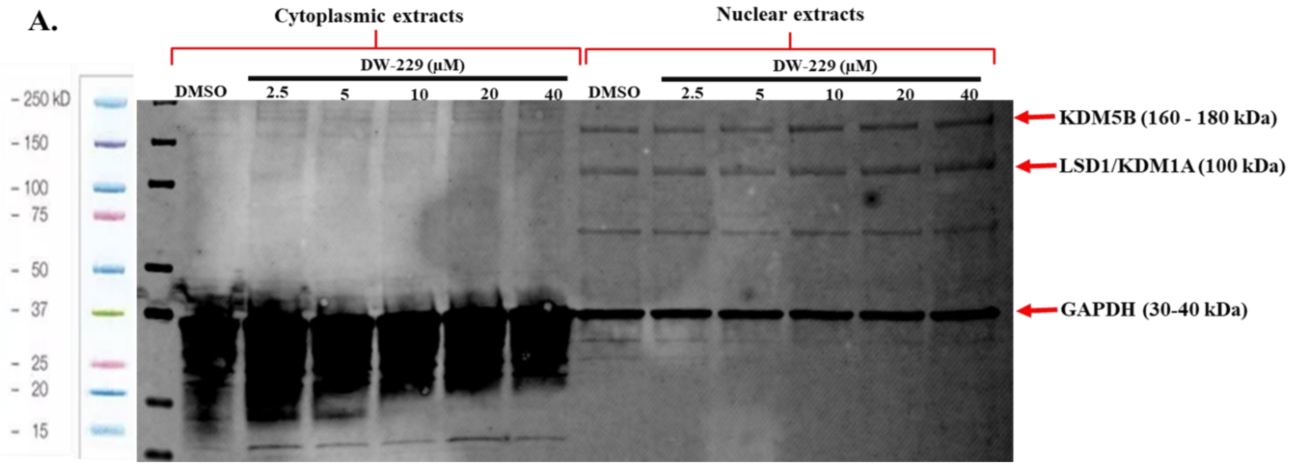

Figure S28. Full immunoblot gel image of MDA-MB-231 cells treated with **DW-229** for 24 h: *KDM5B antibody cocktail.*

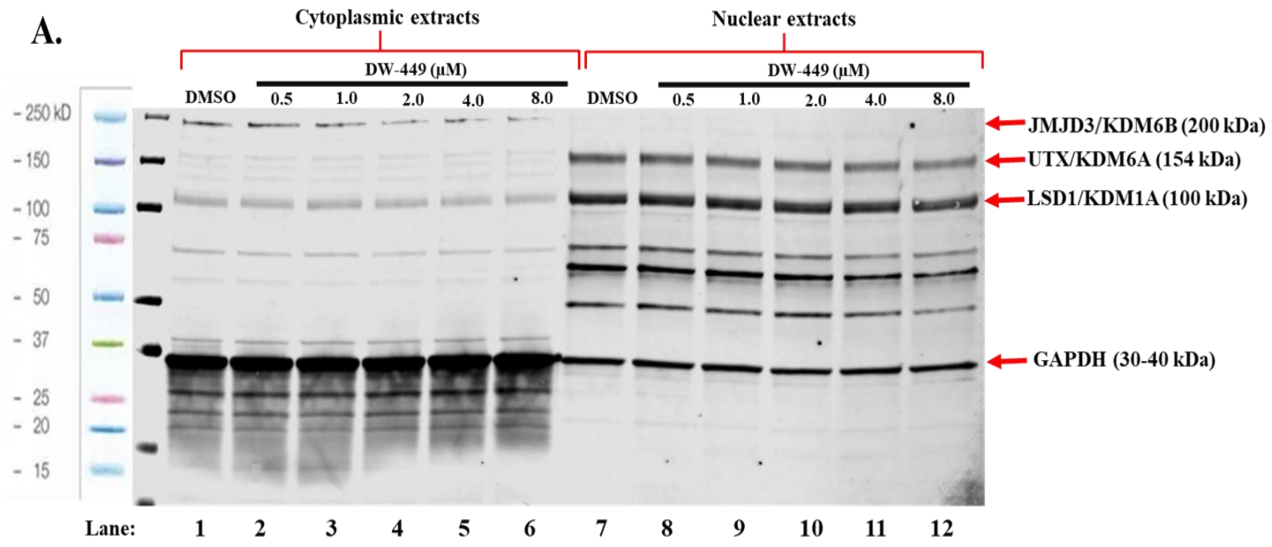

Figure S29. Full immunoblot gel image of MDA-MB-231 cells treated with **DW-449** for 24 h: *KDM1A, KDM6A, and KDM6B antibody cocktail.*

A.

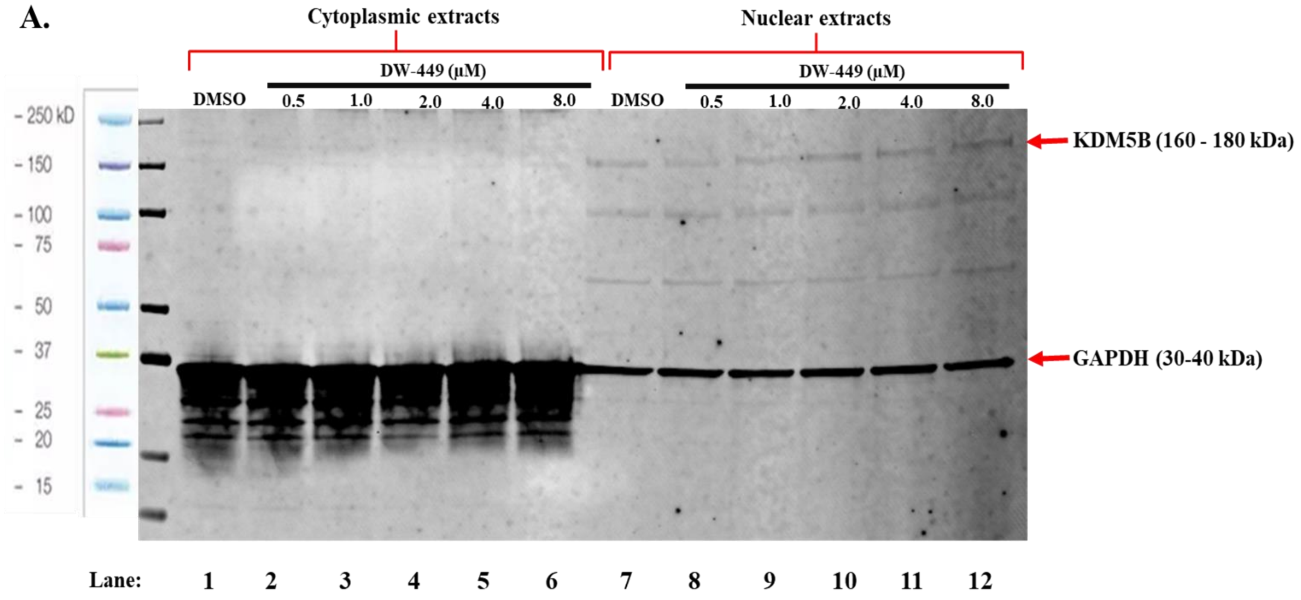

Figure S30. Full immunoblot gel image of MDA-MB-231 cells treated with **DW-449** for 24 h: *KDM5B antibody cocktail.*

Table S3. Effects of **DW-229** relative to DFP on KDM expression in MCF-7 cells. Log2 fold change and p-values highlighting the high significance of KDM2A (p = 0.011, **DW-229**/DFP 2x IC_{50}), KDM3A (p = 2.5e-02, **DW-229**/DFP IC_{50}, p = 9.4e-04, **DW-229**/DFP 2x IC_{50}), and KDM5B (p = 1.8e-04, **DW-229**/DFP IC_{50}, p = 2.9e-07, **DW-229**/DFP 2x IC_{50}). ns = not significant.

	**DW229/DFP IC50**		**DW229/DFP 2xIC50**	
	**log2 fold change**	**p value**	**log2 fold change**	**p value**
KDM1A	-0.3	ns	-0.2	ns
KDM1B	0.2	ns	0.3	ns
KDM2A	-0.5	ns	-0.8	1.1e-02
KDM2B	-0.2	ns	-0.2	ns
KDM3A	-1.0	2.5e-02	-1.5	9.4e-04
KDM3B	-0.2	ns	-0.2	ns
KDM4A	-0.3	ns	-0.5	ns
KDM4B	-0.2	ns	-0.5	ns
KDM4C	-0.1	ns	-0.4	ns
KDM4D	0.0	ns	0.1	ns
KDM4E	0.0	ns	0.0	ns
KDM5A	-0.1	ns	-0.1	ns
KDM5B	-1.2	1.8e-04	-1.7	2.9e-07
KDM5C	-0.4	ns	-0.5	ns
KDM5D	0.1	ns	0.2	ns
KDM6A	-0.1	ns	-0.1	ns
KDM6B	-0.2	ns	-0.5	ns

UTY	0.1	ns	0.1	ns
KDM7A	0.0	ns	0.2	ns
PHF8	-0.1	ns	-0.3	ns
PHF2	-0.1	ns	-0.2	ns
KDM8	0.0	ns	0.0	ns
TET1	0.0	ns	0.0	ns
TET2	-0.7	ns	-0.4	ns
TET3	-0.3	ns	-0.5	ns

NMR Spectra

¹HNMR Spectra compound 2

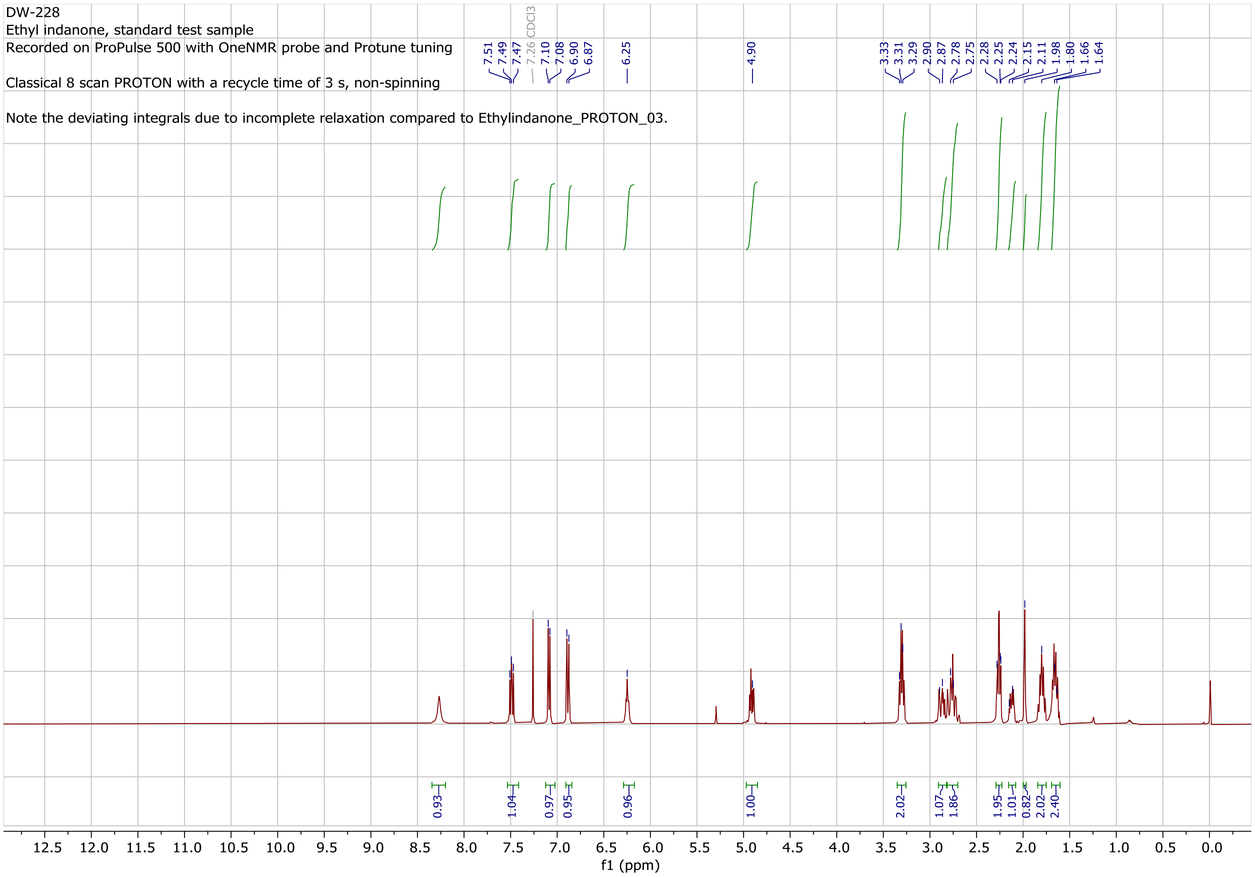

^{1}HNMR Spectra compound 4a

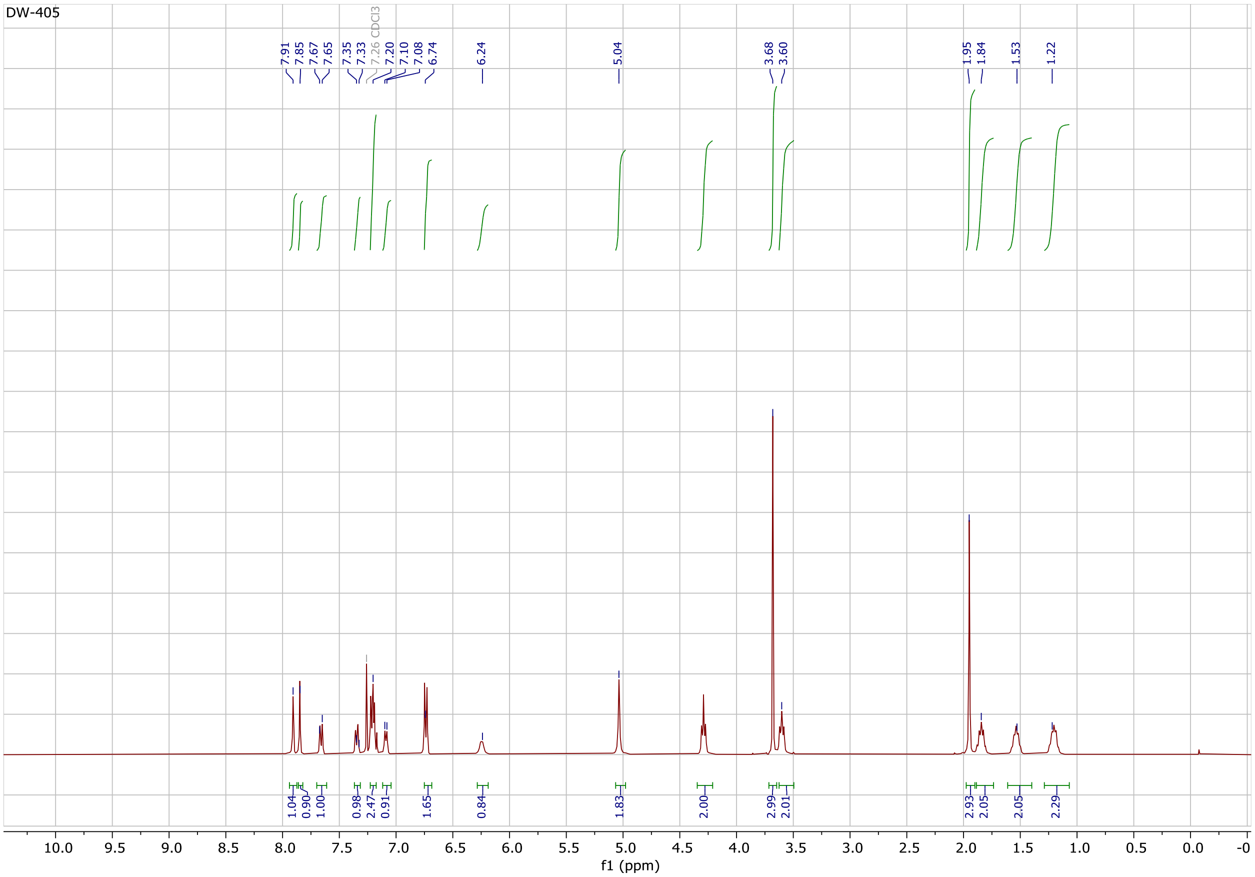

^{1}HNMR Spectra compound 4b

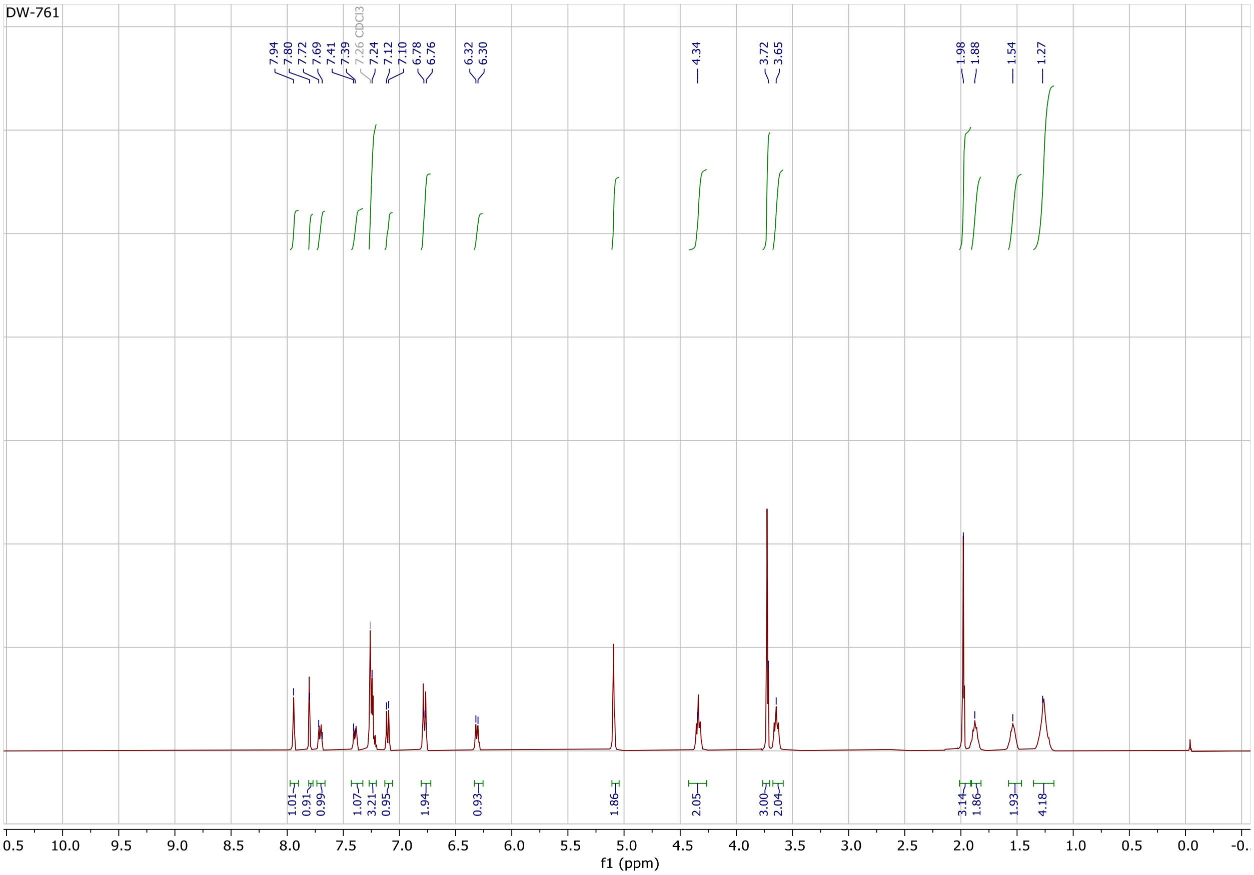

^{1}HNMR Spectra compound 5a

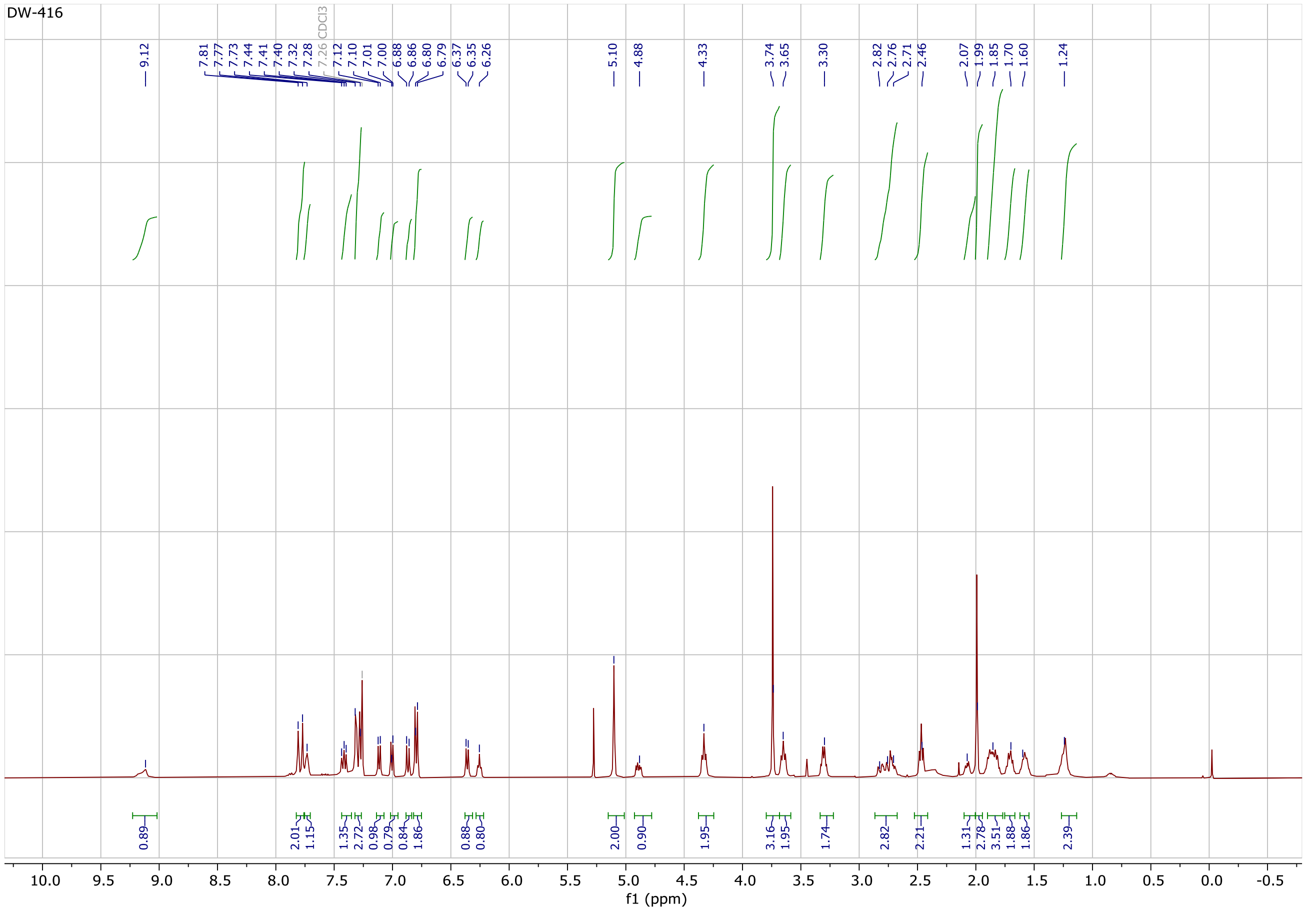

^{1}HNMR Spectra compound 5b

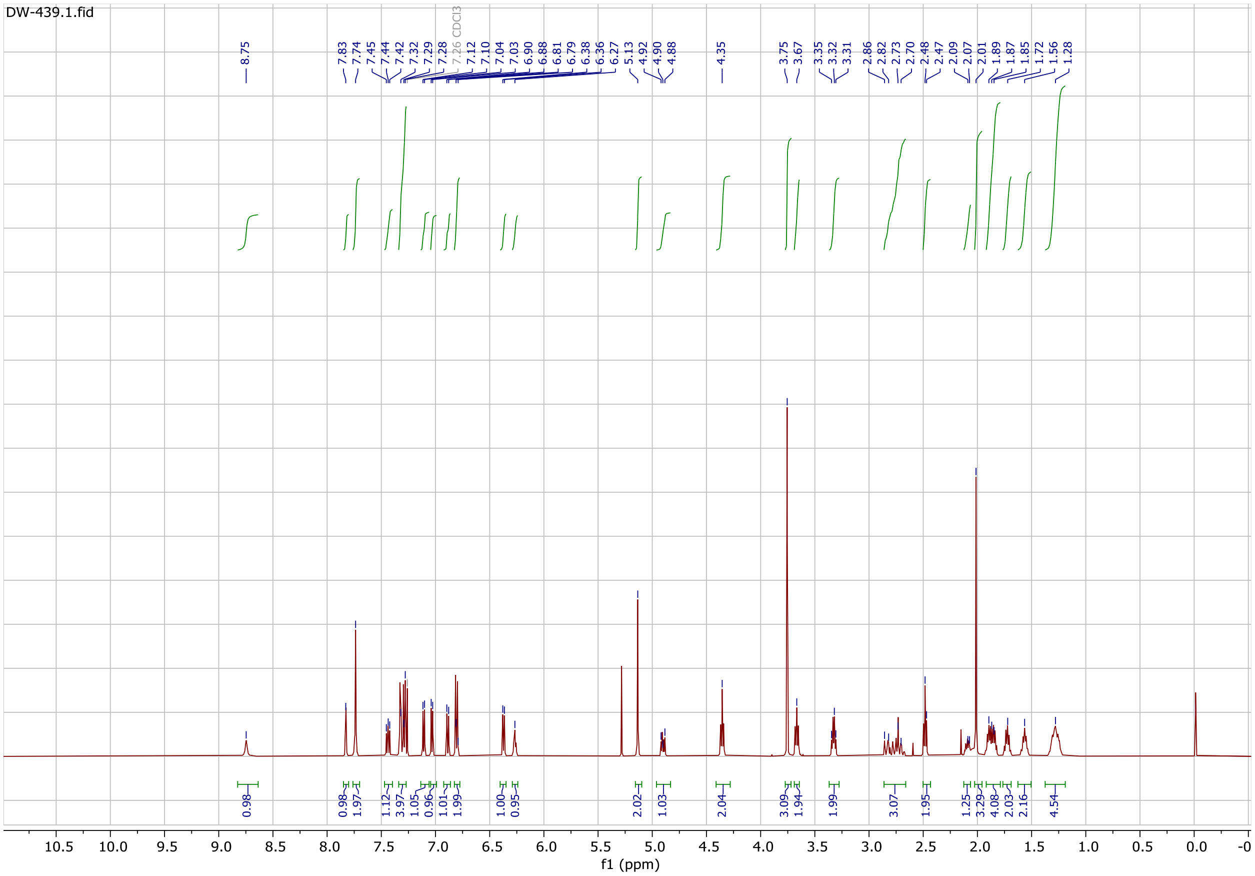

^{1}H and ^{13} C NMR Spectra of compound DW-449

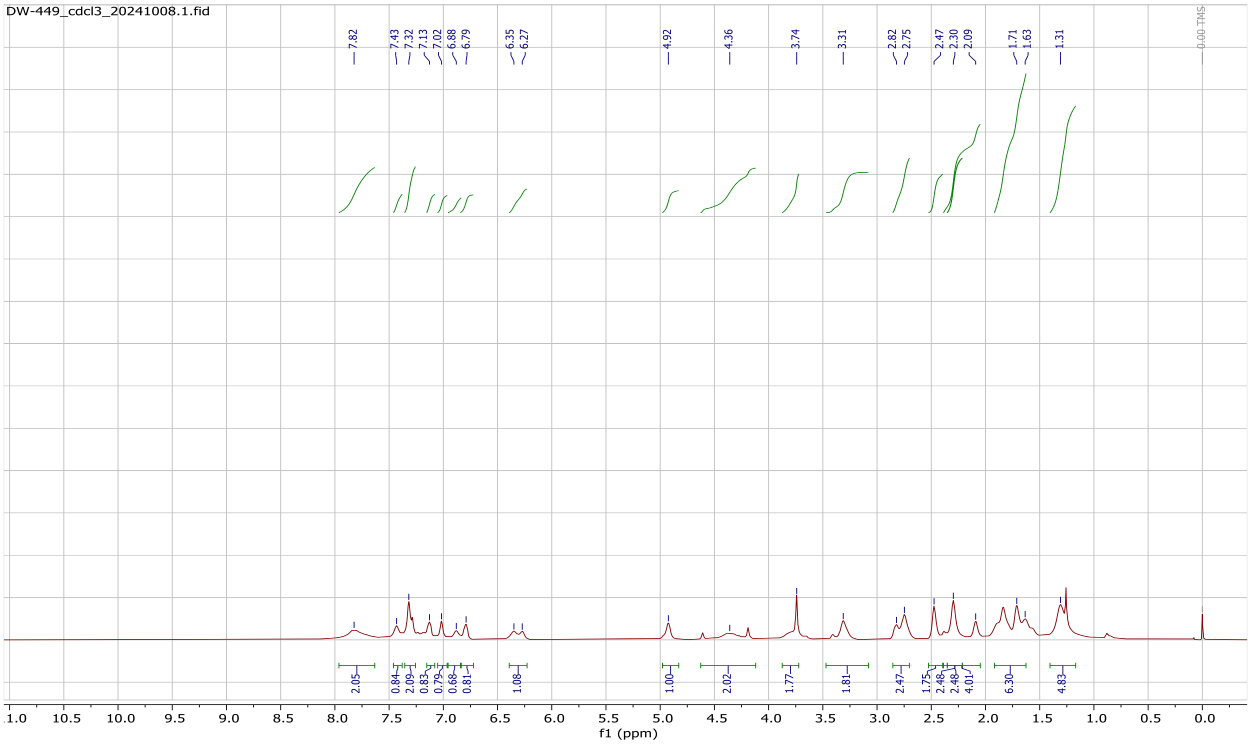

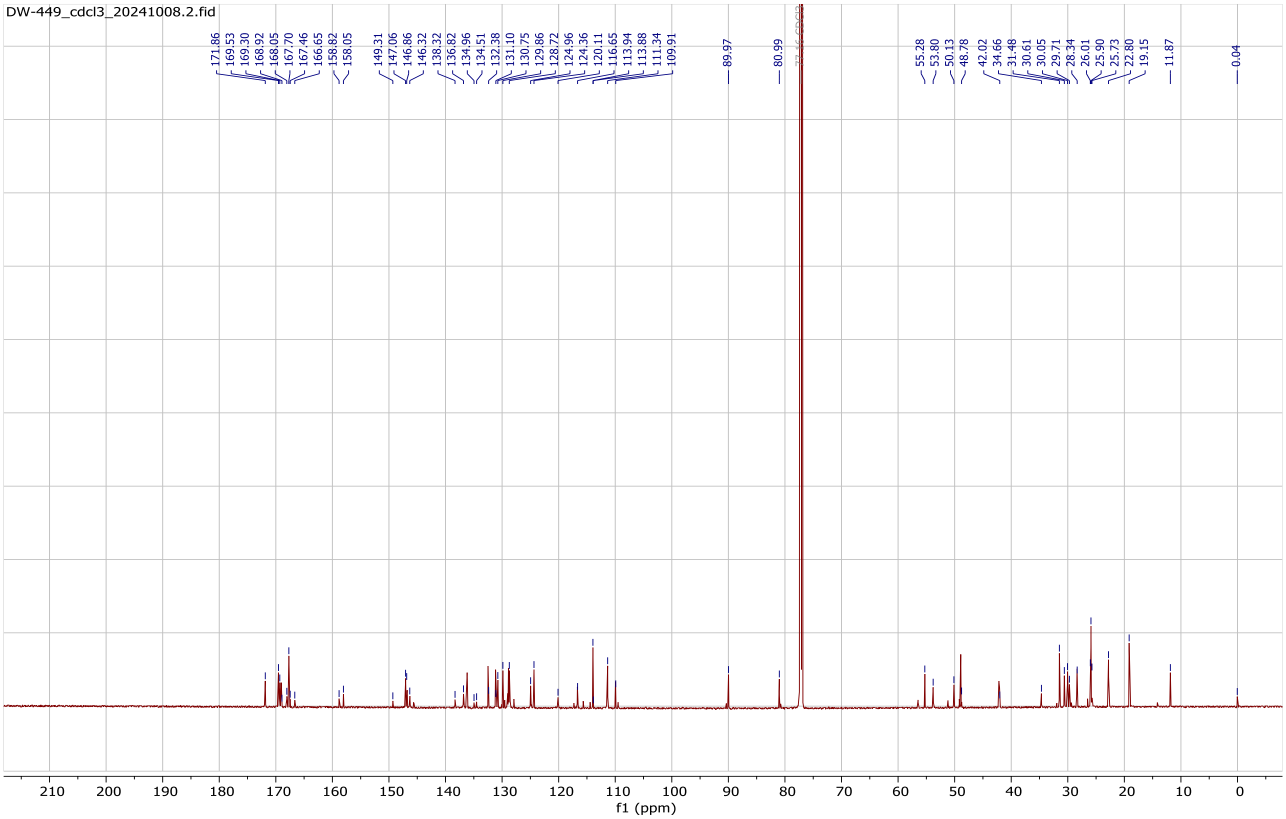

^{1}H and ^{13}CNMR Spectra compound 455

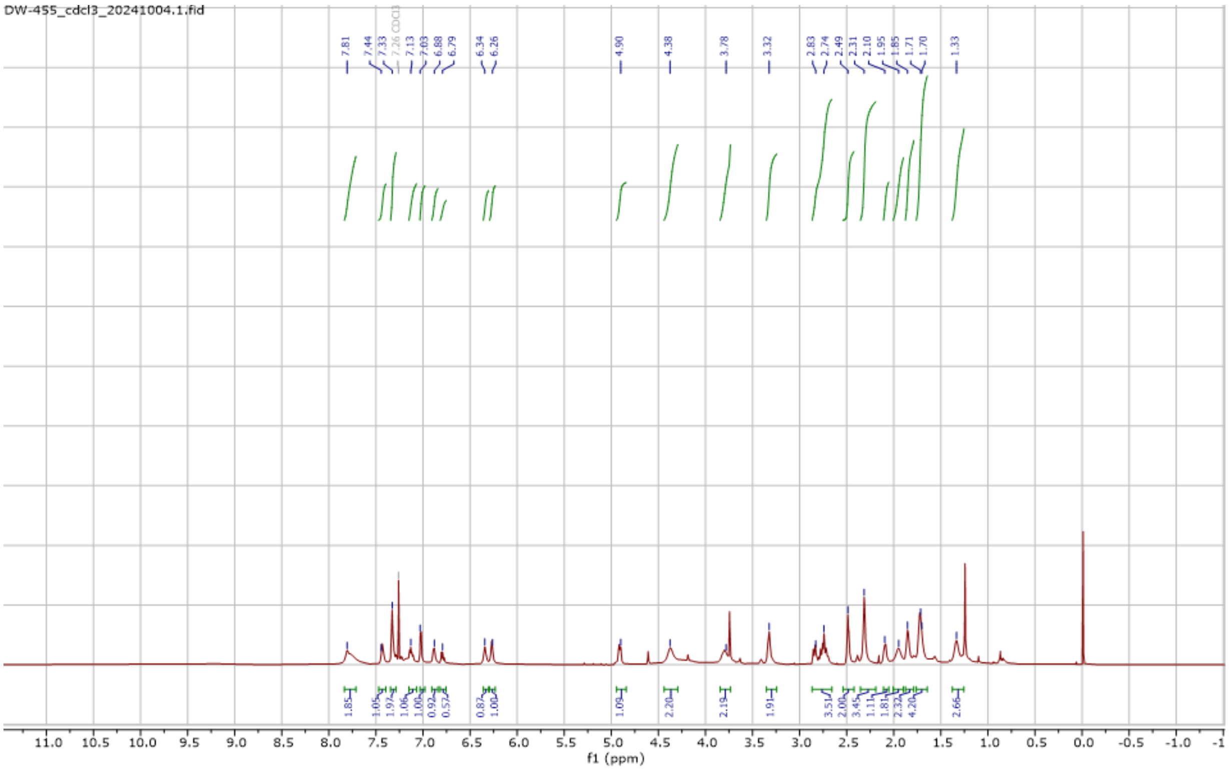

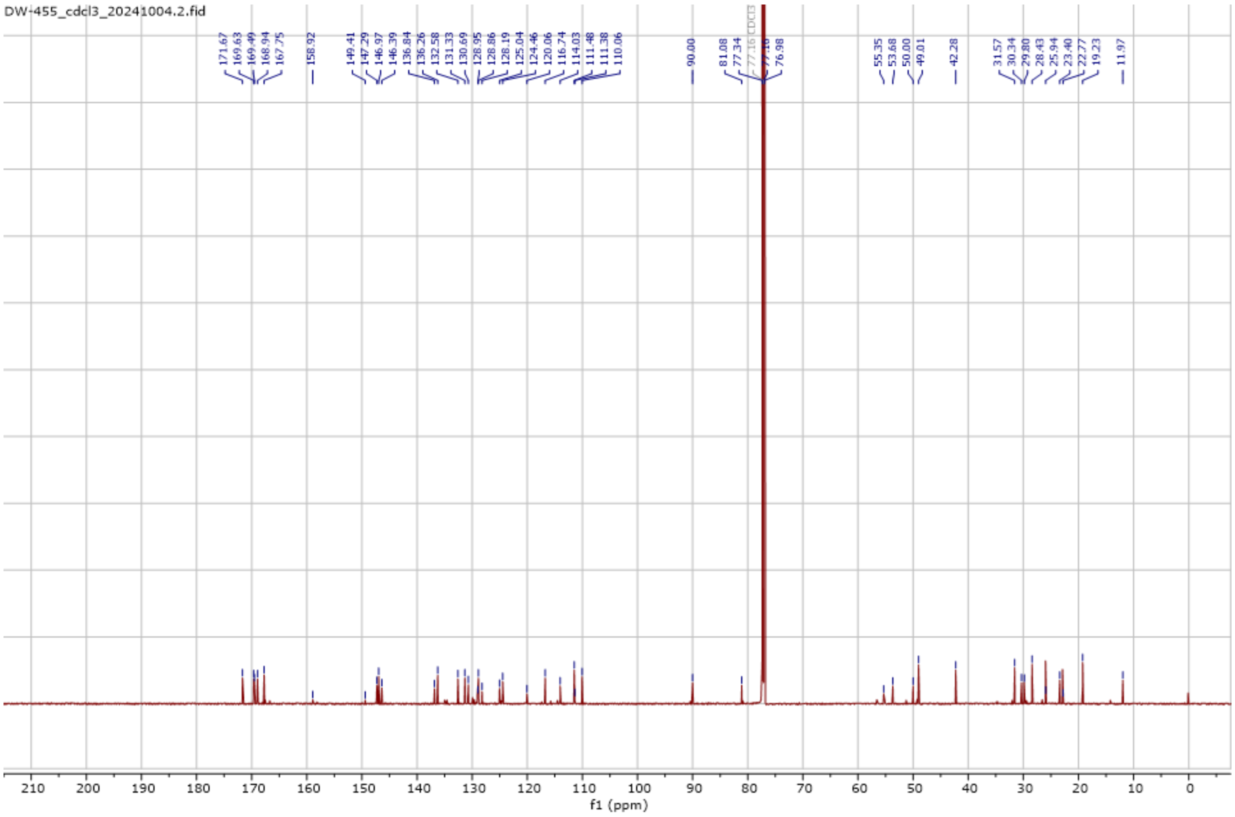

^{1}HNMR Spectra compound 6a

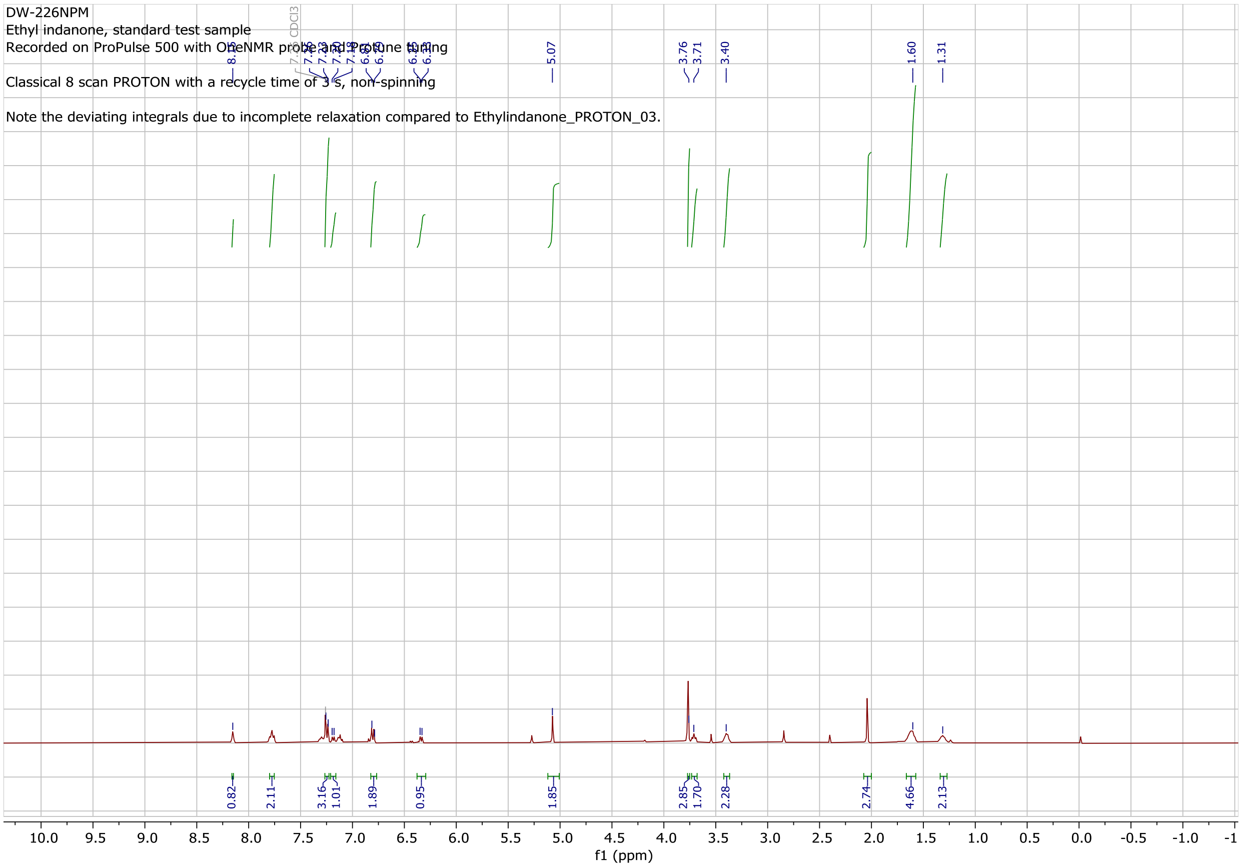

^{1}HNMR Spectra compound 6b

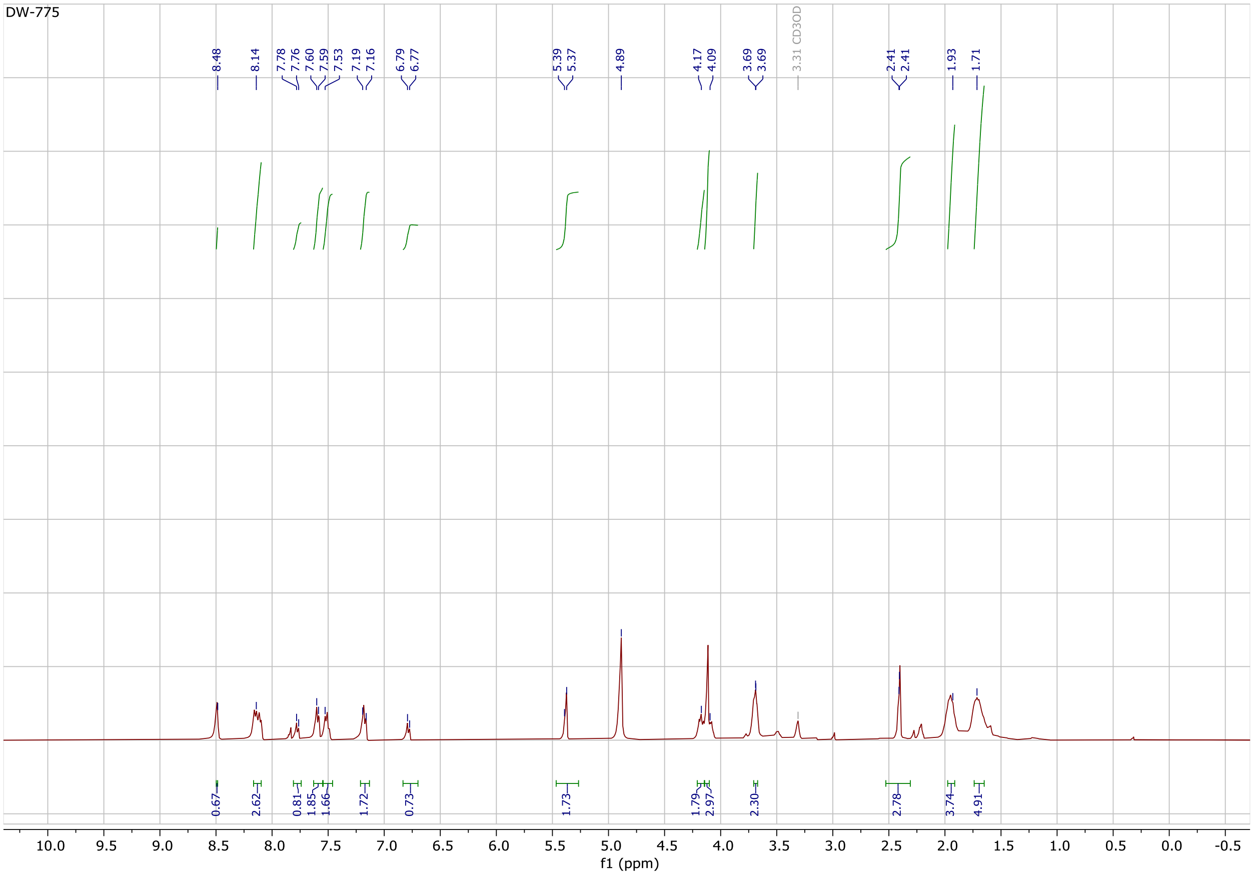

^{1}HNMR Spectra compound 7a

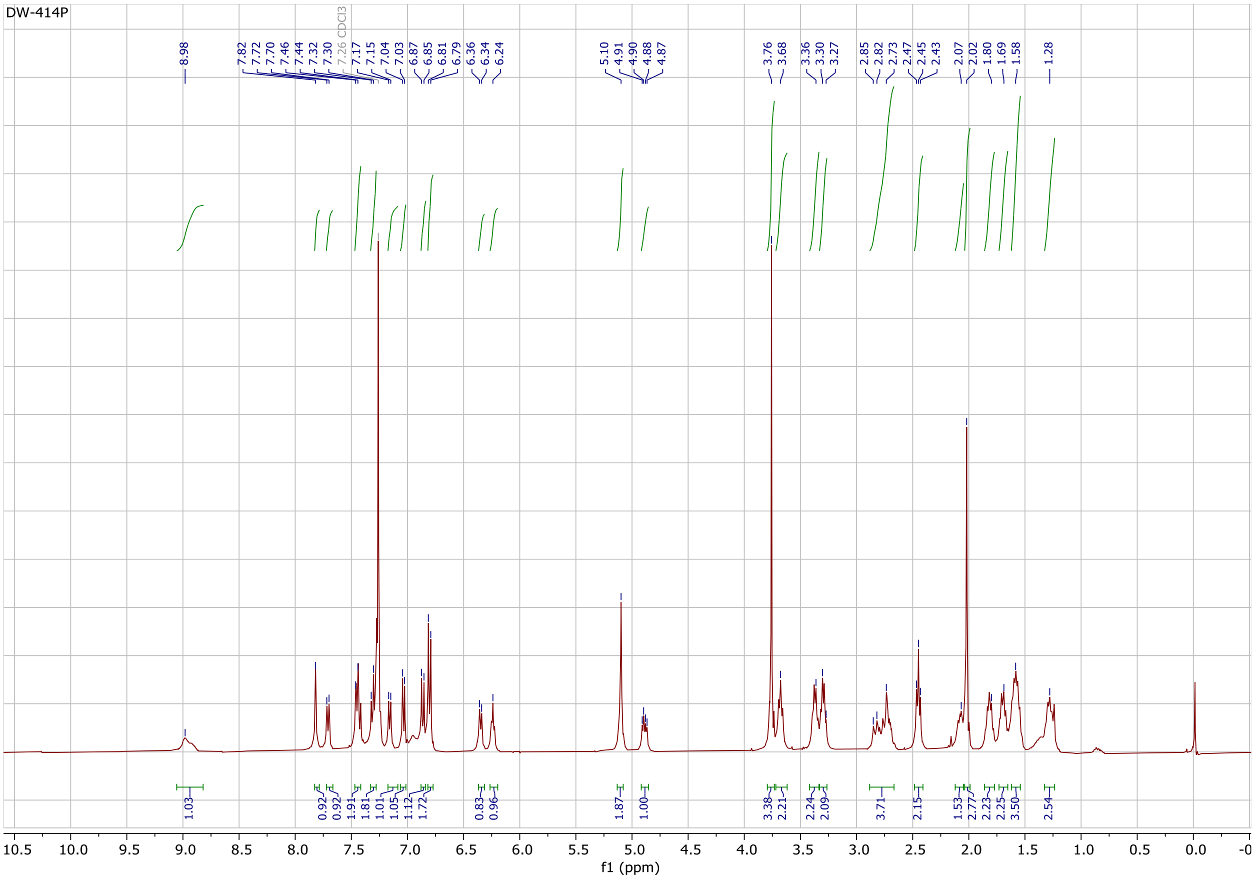

^{1}HNMR Spectra compound 7b

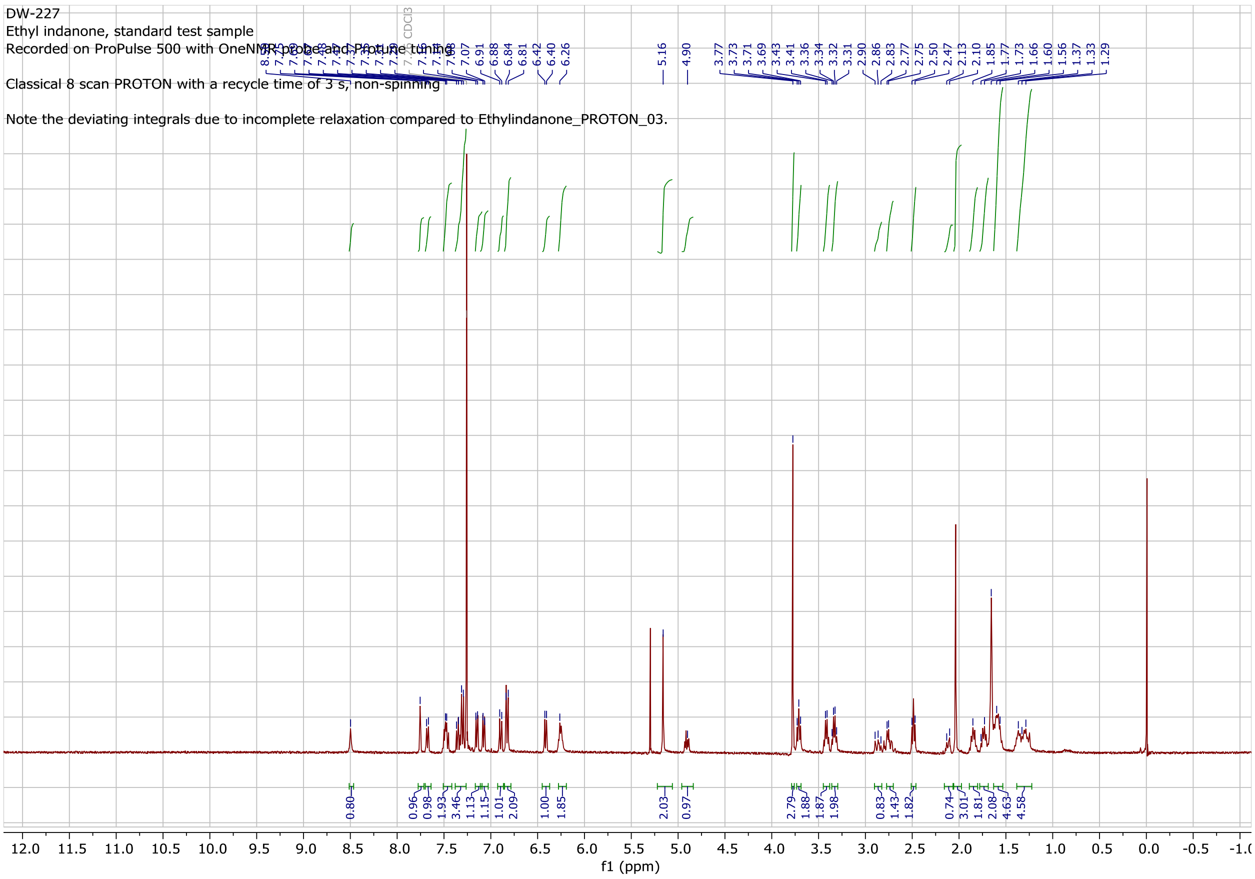

^{1}H and ^{13} C NMR Spectra compound DW-451

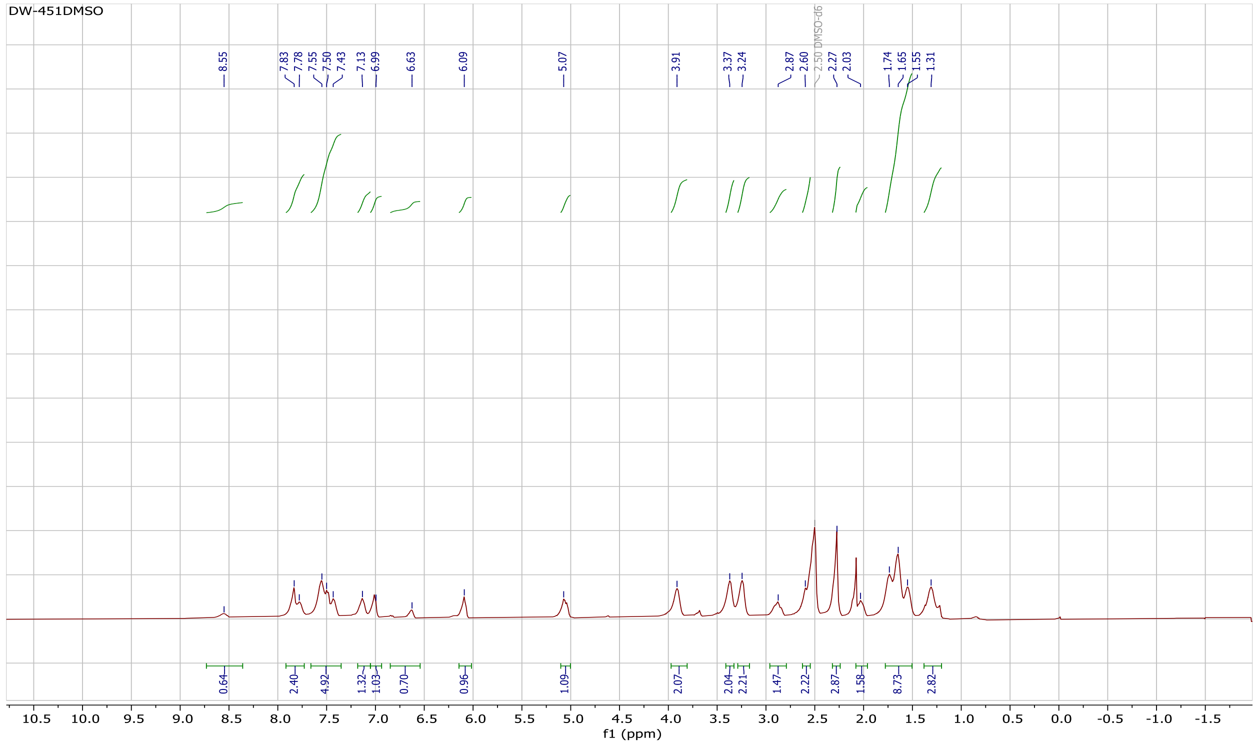

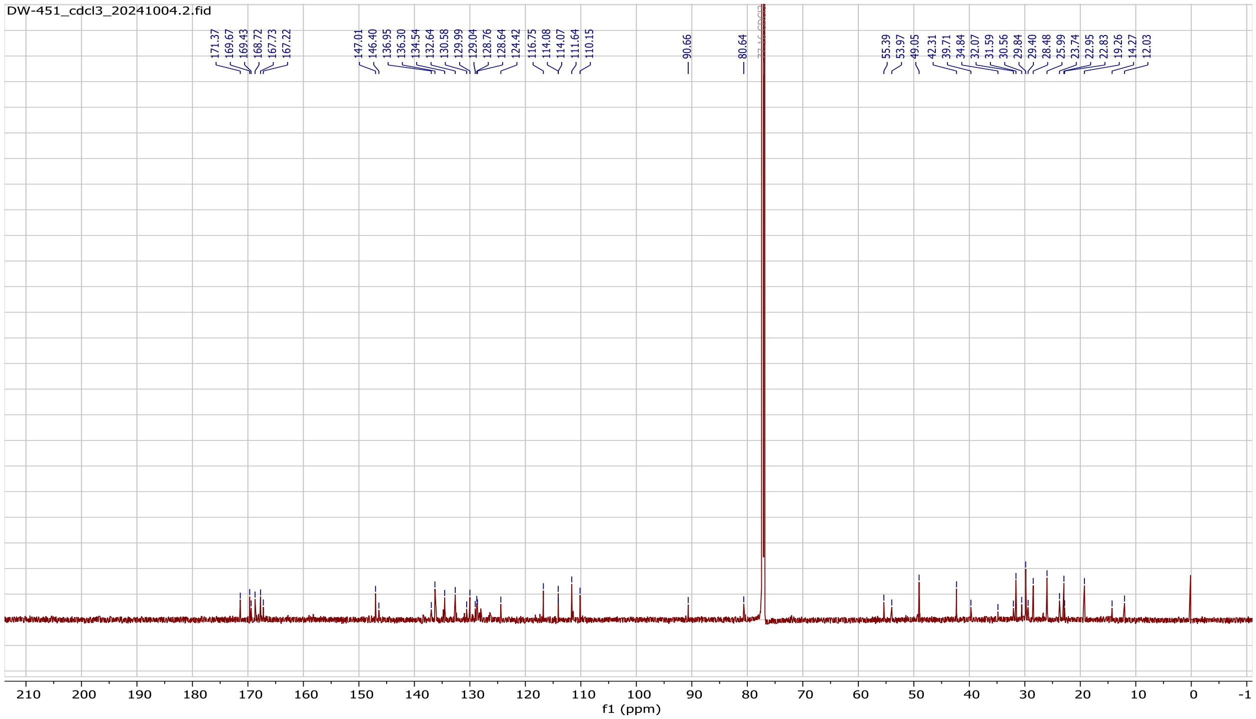

^{1}H and [13] C NMR Spectra compound DW-229compound Purity and UV peak at 254 nm of

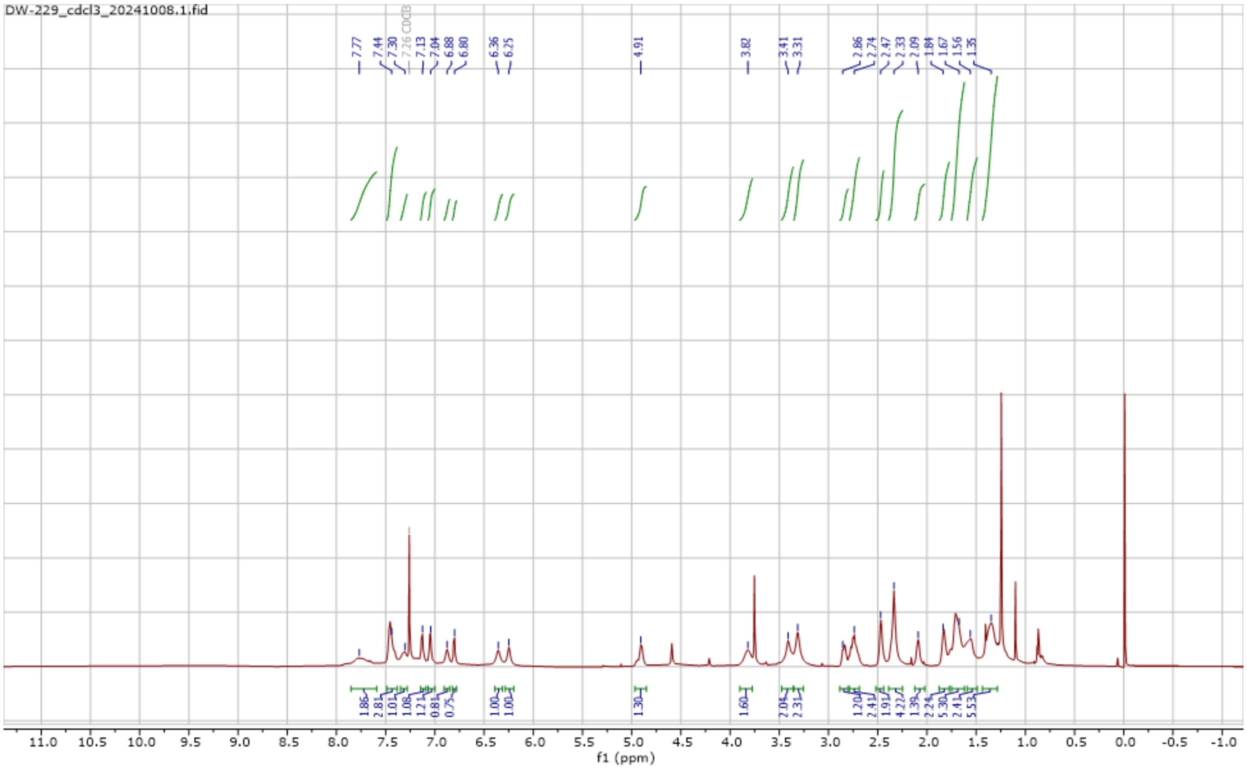

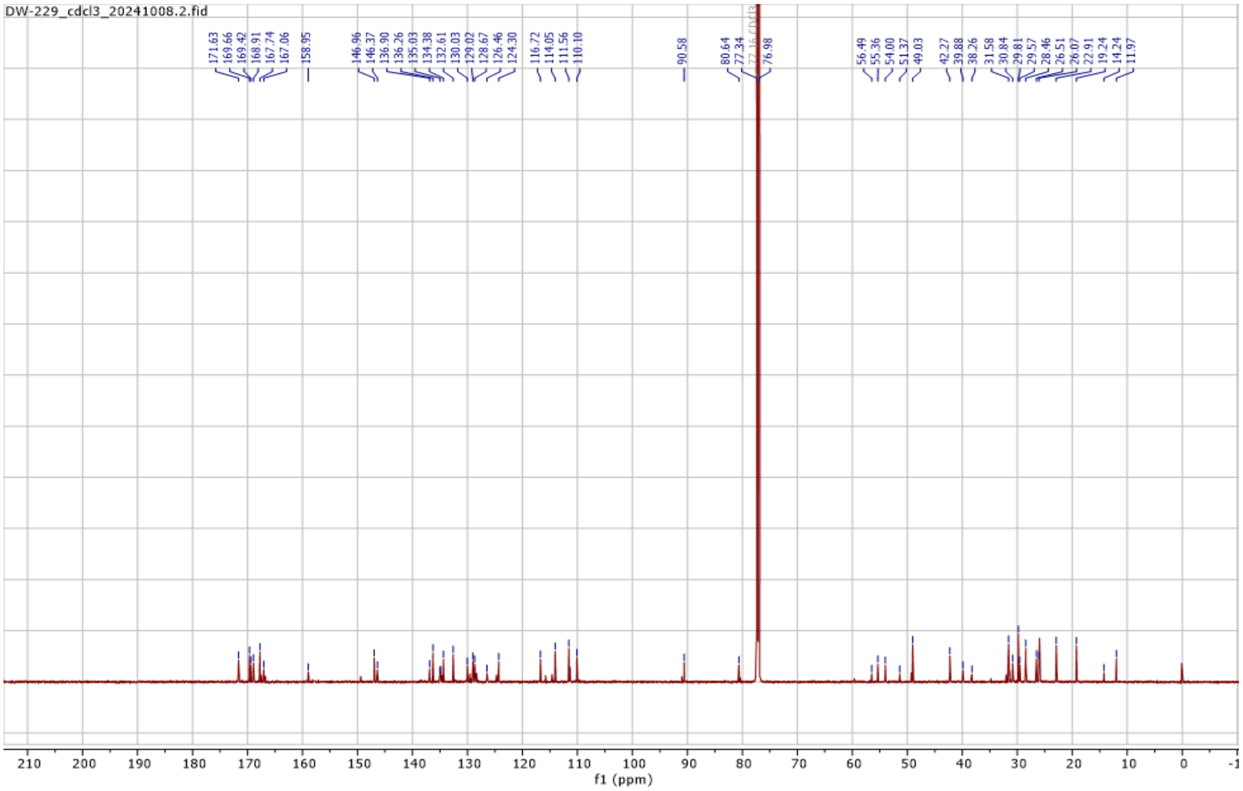

Compound DW-229

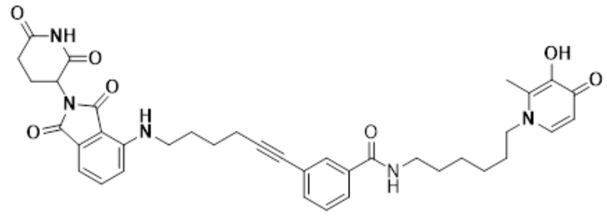

DW-229

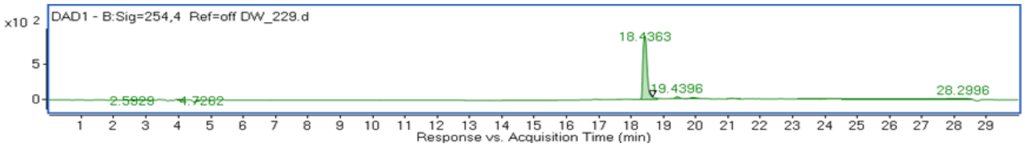

Peaks: + EIC(680.0958-684.6608) Scan

Peak	RT	Area	Height
1	18.5243	3379813411.16	168607720.36
2	19.5503	1117859122.14	53871845.43
4	21.7738	120032781.29	2271651.26
3	21.5001	49990795.16	2549714.22
6	23.9973	37288799.55	2100027.75
5	23.6553	36925788.64	1864098.51

Purity and UV peak at 254 nm of compound DW-449

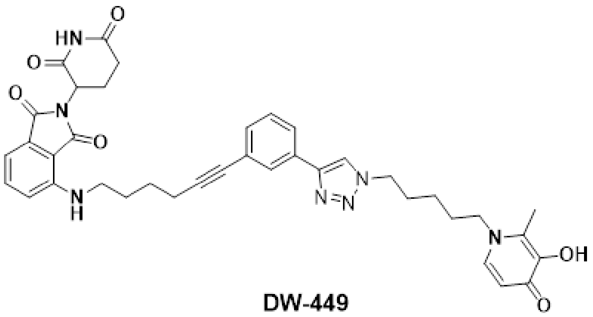

DW-449

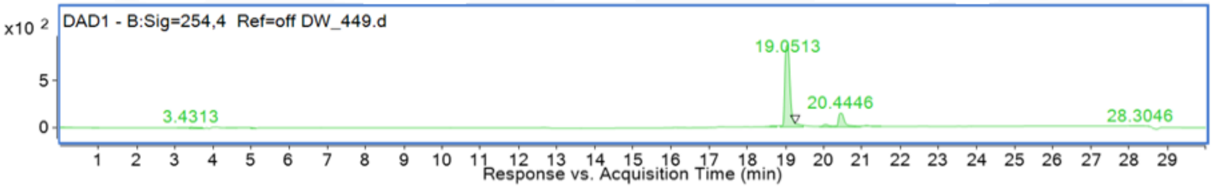

Peaks: + EIC(703.9774-708.4478) Scan

Peak	RT	Area	Height
1	19.1617	2411333887.96	139798601.42
2	20.1538	610623995.02	34884580.88
4	21.8642	86726487.94	1824763.52
6	23.6428	57988427.46	1617430.21
3	21.488	52909962.75	2178642.23
5	22.7536	34819478.69	1742042.86

Purity and UV peak at 254 nm of compound DW-451

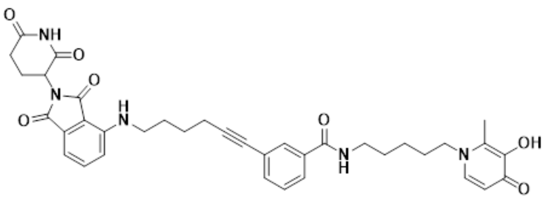

DW-451

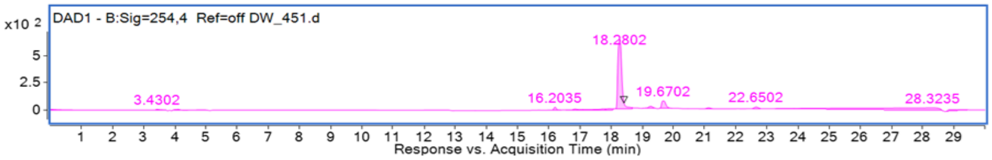

Peak	RT	Area	Height
2	18.3975	2612938983.33	149404464
3	19.355	451419919.81	25626396
1	18.0896	64918475.68	3495341
7	21.7836	48398046.27	1222699
5	20.7918	46923085.71	1720163
4	20.3814	43549586.11	2105039
6	21.4416	37858691.12	1391684
8	22.7414	32852667.56	1202977
9	23.8359	31038000.57	1135780

Purity and UV peak at 254 nm of compound DW-455

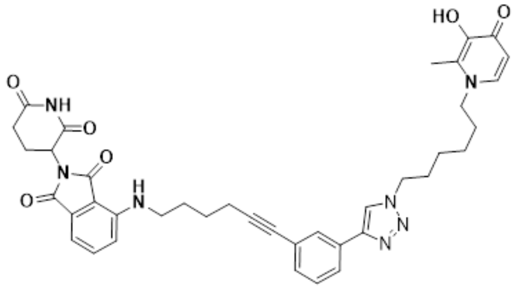

DW-455

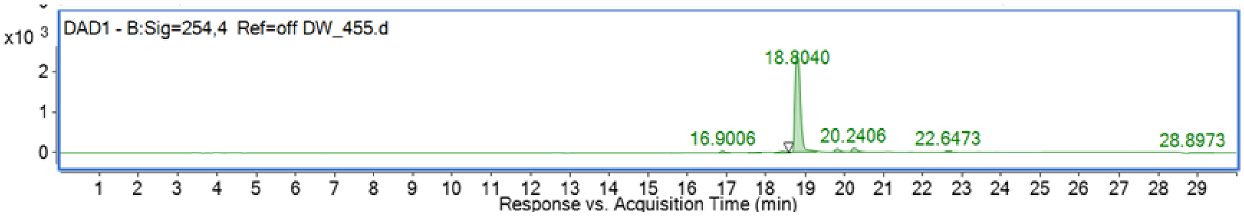

Peak	RT	Area	Height	Typ
2	18.9238	3259521811.32	151964463.1	
3	19.9158	1123848715.62	61894715.63	
4	21.3526	118809808.28	2676815.99	
1	18.5818	80136626.52	6725016.26	
6	22.7892	69001198.22	2427805.06	
7	23.6101	68278078.43	2206780.41	
5	22.3445	40611030.69	1968300.39	
